# A Chirally Locked Bis-perylene Diimide Macrocycle: Consequences for Chiral Self-Assembly and Circularly Polarized Luminescence

**DOI:** 10.1021/jacs.3c13191

**Published:** 2024-02-14

**Authors:** Samuel E. Penty, Georgia R. F. Orton, Dominic J. Black, Robert Pal, Martijn A. Zwijnenburg, Timothy A. Barendt

**Affiliations:** † School of Chemistry, University of Birmingham, Edgbaston, Birmingham B15 2TT, U.K.; ‡ Department of Chemistry, University of Durham, South Road, Durham DH1 3LE, U.K.; § Department of Chemistry, University College London, 20 Gordon Street, London WC1H 0AJ, U.K.

## Abstract

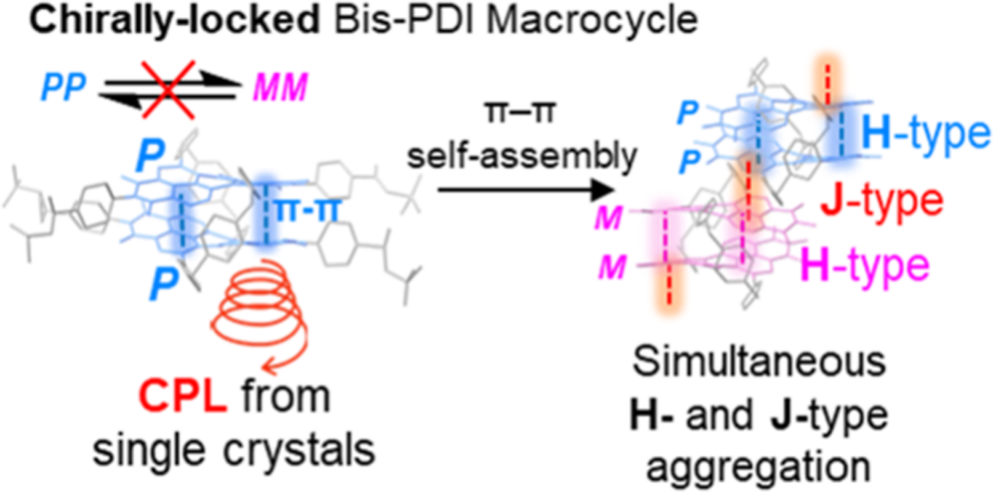

Macrocycles containing chiral organic dyes are highly valuable for the development of supramolecular circularly polarized luminescent (CPL) materials, where a preorganized chiral framework is conducive to directing π–π self-assembly and delivering a strong and persistent CPL signal. Here, perylene diimides (PDIs) are an excellent choice for the organic dye component because, alongside their tunable photophysical and self-assembly properties, functionalization of the PDI’s core yields a twisted, chiral π-system, capable of CPL. However, configurationally stable PDI-based macrocycles are rare, and those that are also capable of π–π self-assembly beyond dimers are unprecedented, both of which are advantageous for robust self-assembled chiroptical materials. In this work, we report the first bay-connected bis-PDI macrocycle that is configurationally stable (Δ*G*^⧧^ > 155 kJ mol^–1^). We use this chirally locked macrocycle to uncover new knowledge of chiral PDI self-assembly and to perform new quantitative CPL imaging of the resulting single-crystal materials. As such, we discover that the chirality of a 1,7-disubstituted PDI provides a rational route to designing H-, J- and concomitant H- and J-type self-assembled materials, important arrangements for optimizing (chir)optical and charge/energy transport properties. Indeed, we reveal that CPL is amplified in the single crystals of our chiral macrocycle by quantifying the degree of emitted light circular polarization from such materials for the first time using CPL-Laser Scanning Confocal Microscopy.

## Introduction

The importance of macrocycles can be traced back to the origins of supramolecular chemistry.^1^ Since then, chiral macrocycles such as cyclodextrins,^2^ cyclopeptides,^3^ and pillararenes^4^ have provided added value due to their chiral recognition^5^ and chiral self-assembly properties.^6,7^ Now, macrocycles containing chiral organic dyes are driving the development of chiroptical materials,^8−11^ including those that exhibit circularly polarized luminescence (CPL),^12,13^ which have burgeoning applications in sensing,^14−16^ security,^17,18^ and optical communications.^14,19−23^ The macrocycle’s preorganized chiral framework is conducive to a strong and persistent CPL signal, which, in tandem with its supramolecular properties, promises efficacious CPL materials through molecular self-assembly.

Alongside molecular design, advancements in CPL materials are also driven by new CPL diagnostics. Here, Circularly Polarized Luminescence-Laser Scanning Confocal Microscopy (CPL-LSCM) is a valuable new technique for imaging CPL materials which combines fast acquisition times and high spatial resolution.^16,26^ A key parameter for CPL materials is the emission dissymmetry factor (*g*_lum_), which represents the degree of circular polarization. While the *g*_lum_ is readily measured in solution using CPL spectroscopy, quantifying CPL dissymmetry in single-crystal materials using CPL-LSCM has yet to be achieved. Furthermore, most organic CPL emitters have low dissymmetry factors (*g*_lum_ ≈ 10^–3^ or lower),^27^ which limits their applicability for CPL imaging and indeed their ultimate practicality.

**Figure 1:**
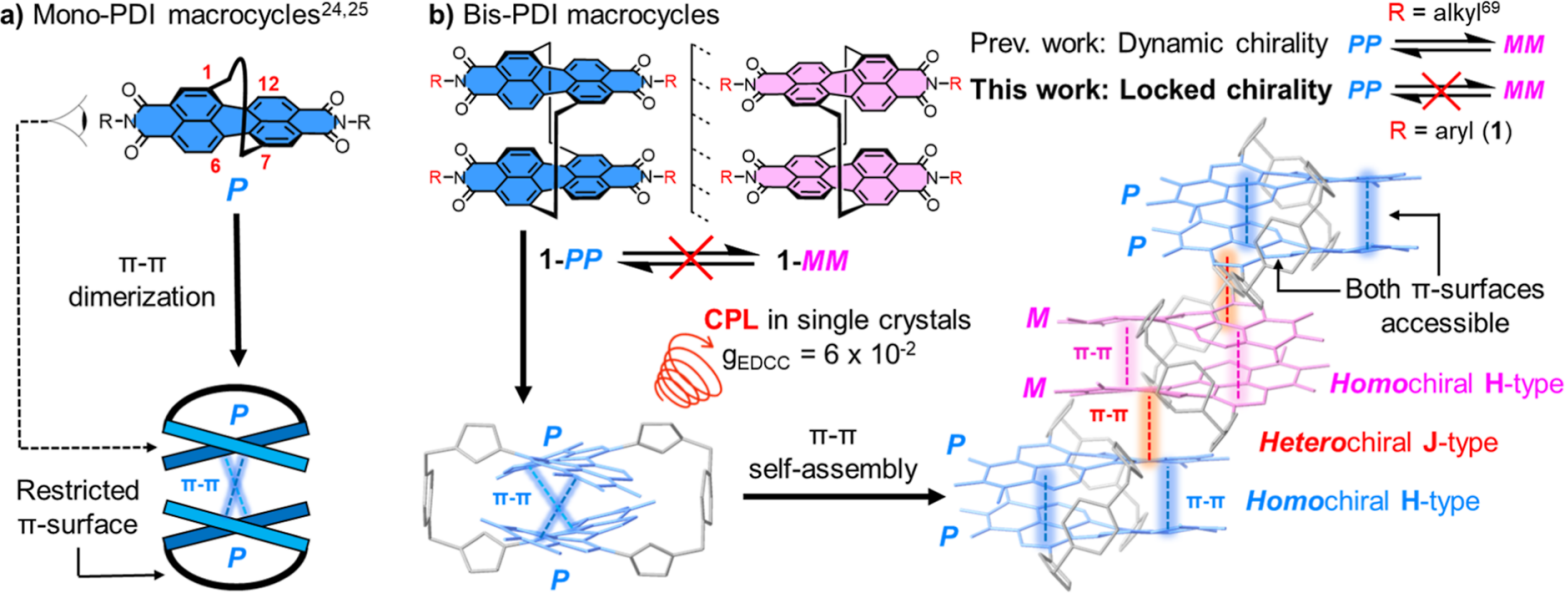
(a) Cartoon of a 1,7-strapped mono-PDI macrocycle and its preference for homochiral dimerization.^24,25^ (b) Chirally locked bis-PDI macrocycle **1** facilitates π–π stacking on both PDI π-surfaces to afford CPL in single crystals and concurrent H- and J-type aggregates.

Perylene diimides (PDIs) are a class of luminescent organic dyes and promising supramolecular building blocks for chiroptical materials.^28−37^ Alongside excellent photophysical properties, PDIs that are functionalized at one or more of their bay positions (1,6,7,12) are chiral due to twisting of the aromatic perylene core, which generates ***M*** or ***P*** atropisomers (Figure 1a).^38^ Core twisted PDIs may undergo self-assembly through π–π interactions,^39−41^ which provides the potential to amplify the *g*_lum_ of CPL due to excitonic coupling between the chiral chromophores.^42,43^ However, CPL measurements on single crystals of PDI dyes are unprecedented.

PDIs most commonly assemble through face-to-face^44^ or slipped-stack^45^ arrangements of their π-surfaces,^46^ known as H- and J-type aggregates respectively, with differences in excitonic coupling leading to blue- and red-shifted absorption spectra, respectively.^47^ The formation of concurrent H- and J-type aggregates is rare for organic materials in general, yet highly advantageous for tuning optoelectronic properties^48−55^ since H-type coupling is conducive to charge carrier mobility,^56,57^ while J-type coupling benefits exciton transport and amplified emission.^58^ By analogy, understanding the self-assembly of chiral dyes,^59−61^ including chiral PDIs,^62−66^ is important for chiral optoelectronic materials. However, the connection between PDI chirality and H-/J-type aggregation is poorly understood, and to the best of our knowledge, concurrent H- and J-type aggregates have not been realized with chiral, core-twisted PDIs in the same material.

Upon aggregation, the π–π interactions between 1,7-disubstituted PDIs are stronger than those between 1,6,7,12-tetrasubstituted PDIs because the former are less twisted.^39^ Moreover, since the 1,7 substituents are directed toward the same face of the PDI (Figure 1a),^39^ disubstituted PDIs possess two different π-surfaces, one more sterically hindered than the other. These π-surfaces would be expected to exhibit distinct π–π interactions and thus self-assembly properties. Importantly, the organic dye must be configurationally stable to assess the impact of optical purity on π–π-directed self-assembly (i.e., to compare racemic and enantiopure materials). Of the handful of configurationally stable disubstituted PDIs reported (Δ*G*^⧧^ > 117 kJ mol^–1^),^24,67,68^ π–π self-assembly is limited to the formation of H-type dimers.^24,25^ This is because macrocyclic strapping via the 1,7 bay positions, a necessity for preventing atropisomer interconversion, restricts π–π interactions to only one of the π-surfaces of the mono-PDI macrocycle (Figure 1a).

A promising strategy for extending π–π self-assembly is to strap the PDI core with a second PDI unit, yielding a bay-connected bis-PDI macrocycle^69−71^ with accessible π-surfaces^72^ on both the interior and exterior of the macrocycle, for *intra*- and *inter*-molecular π–π interactions respectively (Figure 1b). Here, the choice of functional group at the PDI’s terminal imide positions is key because it must be of the correct size and shape to facilitate π–π interactions while also inhibiting the interconversion of the macrocycle’s stereoisomers (***PP***, ***PM***, and ***MM***). However, all current bay-connected bis-PDI macrocycles,^70,71^ including our previous “Pink Box”,^69^ exhibit dynamic chirality since their imide groups do not prevent an intramolecular somersault of the PDI imide heads through the macrocycle cavity (Figure 1b).

Herein, we report the first bay-connected bis-PDI macrocycle that is chirally locked (Δ*G*^⧧^ > 155 kJ mol^–1^, Figure 1b). In combining configurational stability with π-surface accessibility, this macrocycle (**1**) enables us to investigate the impact of chirality on the π–π supramolecular self-assembly of 1,7-disubstituted PDIs beyond dimers. We discover that the self-assembly of macrocycle **1** through intermolecular π–π interactions on sterically hindered π-surfaces requires a slipped-stack and heterochiral relationship between PDI units (i.e., ***M***–***P***), while *intra*molecular π–π stacking on the remaining π-surfaces is face-to-face and homochiral (i.e., ***M***–***M*** or ***P***–***P***). Therefore, we show for the first time that the chirality of twisted PDIs can provide a rational route to direct H- and J-type aggregation in the same material. Indeed, the racemate of macrocycle **1** self-assembles into a material containing concurrent intramolecular H- and intermolecular J-type coupling (Figure 1b). Configurational stability enables us to perform CPL-LSCM imaging on both enantiopure and racemic single crystals of macrocycle **1** and so, for the first time, quantify their degree of emitted light circular polarization by calculating an Enantioselective Differential Chiral Contrast dissymmetry factor (*g*_EDCC_), a value akin to a *g*_lum_.

## Results and Discussion

### Macrocycle Design, Synthesis, and Characterization

**Figure 2:**
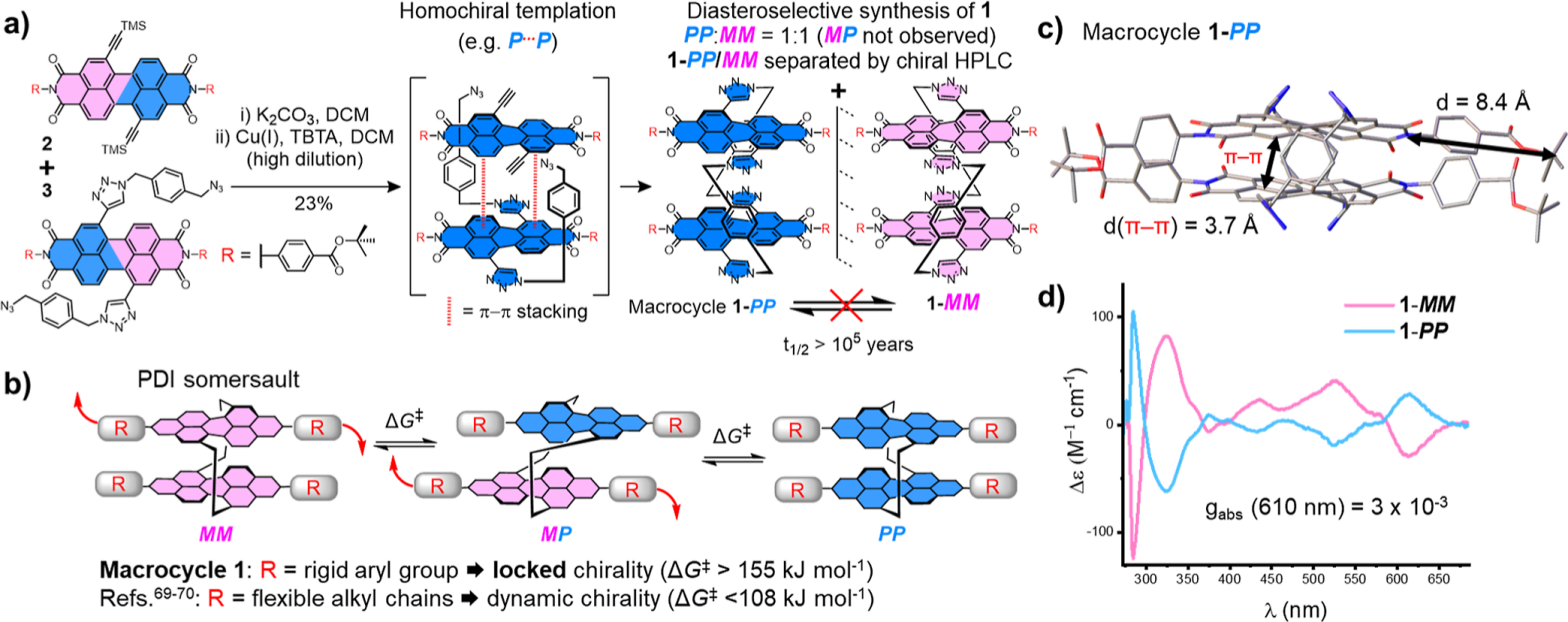
(a) The diastereoselective synthesis of macrocycle **1**. (b) A cartoon of the “intramolecular somersault” mechanism for the interconversion of bis-PDI macrocycle stereoisomers,^70^ which is inhibited in **1**. (c) X-ray crystal structure of macrocycle **1-***PP*. (d) Circular dichroism (CD) spectra of **1-*****MM*** and **1-*****PP*** (10 μM, toluene, and 298 K).

A key element in our design of bis-PDI macrocycle **1** is the use of *tert*-butyl benzoate substituents at the imide termini of the PDI units (Figure 2a,c). We envisaged these substituents would enforce configurational stability on the macrocycle because, from our initial molecular modeling study (Supporting Information, Section S8), these rigid substituents elongate the PDIs by ∼8 Å (Figure S34), thereby preventing them from somersaulting through the macrocycle cavity (*d* = 3.7 Å, Figure 2b,c). Furthermore, the absence of ortho substituents on the phenyl rings is designed to facilitate strong π–π stacking between PDI units, critical for supramolecular self-assembly.

For the final macrocyclization step, macrocycle **1** was synthesized using copper(I)-catalyzed azide–alkyne cycloaddition (CuAAC) “click” chemistry (Figure 2a) and purified by preparatory thin-layer chromatography (TLC). Since the chirality of the bis-PDI macrocycle is locked (vide infra), we expected to obtain all macrocycle stereoisomers from the TLC plate, namely the pair of enantiomers (**1-*****MM***/***PP***) and the heterochiral diastereomer (**1-**MP). However, only **1-*****MM*** and **1-*****PP*** were isolated, which is intriguing, because this means that the macrocyclization reaction is diastereoselective. The exclusive formation of homochiral macrocycles was confirmed by chiral high performance liquid chromatography (HPLC) analysis of the crude reaction mixture, which revealed two macrocycle peaks of equal intensity in the chiral chromatograms corresponding to **1-*****MM*** and **1-*****PP*** (Figure S4). This pair of enantiomers have a single set of signals in their ^1^H NMR spectrum (Supporting Information, Section S1) and, upon their resolution by chiral HPLC, exhibit mirror image circular dichroism (CD) spectra (Figures 2d, S9 and S10), and specific optical rotations [α]_D_^20^ of +1333 and −1333° for **1-*****MM*** and **1-*****PP*** respectively. The enantiomers were assigned by X-ray crystallography and by comparing experimental and time dependent-DFT predicted CD spectra (Sections S3, S4, and S8). Therefore, the configurational stability of **1** enables us to determine the stereochemical outcome of this bis-PDI macrocyclization and indicates the potential for PDI–PDI homochiral templation during the ring–closing reaction (vide infra). Macrocycle **1** was fully characterized by ^1^H and ^13^C NMR spectroscopy, as well as high resolution mass spectrometry and single crystal X-ray crystallography (Sections S1 and S3).

To demonstrate the configurational stability of macrocycle **1**, the **1-*****MM*** enantiomer was heated at 180 °C for 24 h in 1,2-dichlorobenzene and, when subsequently reinjected back onto the chiral HPLC column, the chromatogram was unchanged (Figures S2 and S3). Therefore, the free energy barrier to macrocycle stereoisomer interconversion (Δ*G*^⧧^) is at least 155 kJ mol^–1^,^73^ with a racemization half-life (*t*_rac_^1/2^) of more than 10^5^ years at room temperature.^74^ This Δ*G*^⧧^ is significantly higher than for all previously reported bay-connected bis-PDI macrocycles (Δ*G*^⧧^ < 108 kJ mol ^–1^), which have *t*_rac_^1/2^ values ranging from minutes to days.^69,70^ The configurational stability of macrocycle **1** provides excellent evidence for the “intramolecular somersault” mechanism since the PDI is now too long to flip through the cavity (Figure 2b), as seen from the crystal structure (Figure 2c). Alongside single crystals of the macrocycle racemate (**1-***rac*), the configurational stability of **1** also enabled us to grow enantiopure single crystals of **1-*****PP*** and **1-*****MM*** without the risk of forming scalemic or racemic mixtures during the crystallization process (vide infra). Single crystals of **1-***rac* were grown by the slow diffusion of methanol into a chloroform solution, while the growth of **1-*****PP***/***MM*** crystals required the slow diffusion of hexane into a 1:1 chloroform:1,2-dicholobenzene solution.

### Supramolecular Self-Assembly

**Figure 3:**
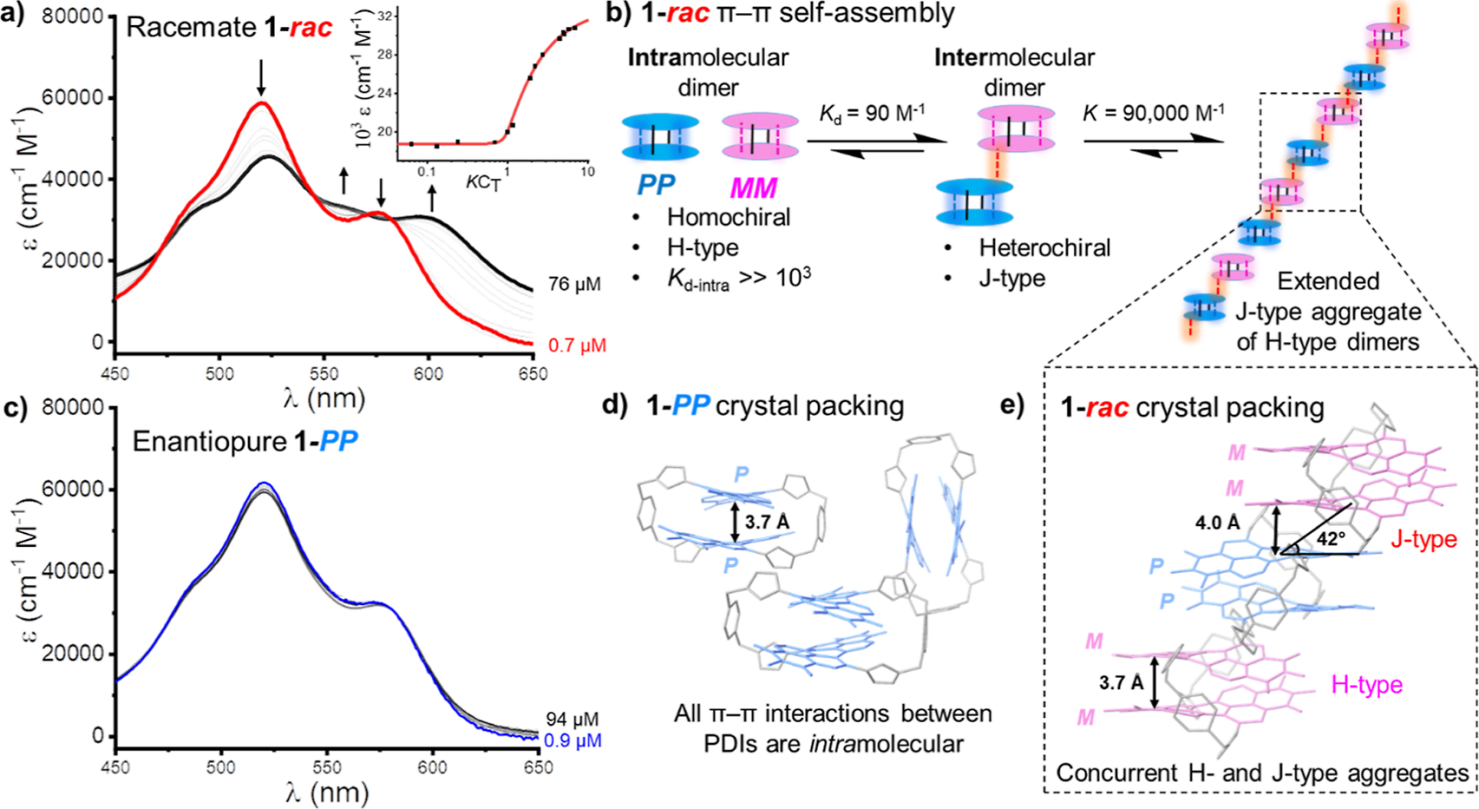
(a) UV–vis spectrum of **1-***rac* upon increasing the concentration (3:2 CH_2_Cl_2_:*n*-hexane, 298 K). Inset: black squares are the change in ε (λ = 596 nm) for the titration in (a). Red line is the binding isotherm from a cooperative nucleation-elongation model. (b) Schematic of the π–π self-assembly of **1-***rac*. (c) UV–vis spectrum of **1-*****PP*** upon increasing the concentration (3:2 CH_2_Cl_2_:*n*-hexane, 298 K). (d) Packing of **1-*****PP*** in the X-ray crystal structure. (e) Packing of **1-***rac* in the X-ray crystal structure.

The configurational stability of **1** provides us with the opportunity to understand how the chirality of 1,7-disubstituted PDIs impacts their supramolecular self-assembly. Therefore, with enantiopure (**1-*****MM*** and **1-*****PP***) and racemic (**1-***rac*) samples in hand,^75^ we investigated macrocycle self-assembly in solution by UV–vis spectroscopy because of the characteristic electronic absorptions of PDI aggregates. At relatively low concentrations (down to 1 μM), the UV–vis spectra of **1-***rac* and **1-*****PP*** are identical (Figure 3a,c and Table 1) and diagnostic of H-type aggregation^46^ since, relative to the PDI monomer **3** (Figure S15, Table 1), λ_max_ of the PDI S_0_–S_1_ absorption band is blue-shifted (Δλ = 30 nm) and the vibronic ratio of this band is reduced (ε_0–0_/ε_0–1_ = 0.54 vs 1.14) with an exciton coupling energy of *J* = 420 cm^–1^ (Section S5b).^76,77^ This is due to the formation of an intramolecular homochiral PDI π–π dimer, as seen in the crystal structure (Figure 2c). In contrast to our previous bis-PDI macrocycle,^69^ the homochiral dimer in **1** persists in chlorinated solvents such as dichloromethane and 1,1,2,2-tetrachloroethane (TCE), solvents that are considered the most competitive for PDI π–π stacking interactions (Figure S13).^78,79^ Importantly, the intramolecular homochiral π–π stacking in dichloromethane (Figure S14) provides excellent evidence for homochiral π–π templation during the macrocycle synthesis in the same solvent and hence a probable explanation for the observed diastereoselectivity (vide supra).

**Table 1:** Photophysical and Chiroptical Properties of Enantiopure (**1-*****MM***/***PP***) and Racemic (**1-***rac* and **3**) Compounds in a Toluene Solution

compound	abs. λ_max_ (nm)^a^	em. λ_max_ (nm)^a^	Φ (%)^b^	τ (ns)^c^	|*g*_abs_| at 610 nm^a^	|*g*_lum_| at 675 nm^a^
**1-***rac*	522	635	35	36^d^		
**1-*****MM***/***PP***	522	635			3 × 10^–^^3^	2 × 10^–^^2^
**3 (racemic)**	552	600	70	8^e^		

a Concentration = 10 μM.

b Concentration = 1 μM.

c Concentration = 5 μM.

d λ_ex_ = 373 nm, λ_em_ = 635 nm.

e λ_ex_ = 373 nm, λ_em_ = 604 nm.

Since we could not disrupt the intramolecular π–π stacking in macrocycle **1**, we used acyclic bis-triazole PDI **3** to estimate the binding strength of the PDI dimer (*K*_d_). Of course, the *K*_d_ measured with **3** provides a lower estimate of *K*_d_ in **1**, since the former is an intermolecular system (i.e., *K*_d-inter_) which is less preorganized than macrocycle **1** (i.e., *K*_d-intra_). Upon concentration of acyclic PDI **3** up to 418 μM in 3:2 CH_2_Cl_2_:*n*-hexane solution (Figure S22) the monomeric UV–vis spectrum evolves into one characteristic of H-type aggregation (ε_0–0_/ε_0–1_ decreases from 1.14 to 0.74), from which we calculated a *K*_d-inter_ = 1677 M^–1^ by nonlinear curve fiting to the monomer–dimer model (Figure S23).^80^ We note that the magnitude of the error in this *K*_d-inter_ value (±19%) arises because further changes to the UV–vis spectrum at higher concentrations of **3** are indicative of the formation of larger aggregates than dimers.

While we could not fully decouple PDI dimerization from subsequent binding events using acyclic PDI **3**, bis-PDI macrocycle **1** is ideally suited to investigating chiral π–π self-assembly beyond the dimer limit. This is because macrocycle **1** is preorganized into an intramolecular H-type dimer and has two accessible π-surfaces on its exterior. Notably, while the macrocycle racemate exhibits Beer–Lambert behavior at low concentrations, **1-***rac* shows significant deviations above 10 μM (Figures 3a, S24, and S25). Upon concentration, a new PDI absorption band at λ = 596 nm appears with isosbestic points (λ = 472, 545, 571, 582 nm), indicating the macrocycle “monomer”, in fact, an intramolecular dimer, is in equilibrium with a new aggregated species (Figure 3a). That this absorption band is significantly red-shifted relative to the monomer of acyclic PDI **3** (Δλ = 46 nm) indicates the self-assembly of macrocycle **1-***rac* into a J-type aggregate, which was confirmed through analysis of packing in single crystals (vide infra).

To estimate the binding constant(s) for macrocycle self-assembly, we fitted the data from the concentration-dependent UV–vis spectra to several binding models suited to the aggregation of π-conjugated molecules.^80^ This included models for the formation of larger aggregates, namely the isodesmic model, in which all binding events have the same *K* value, and the modified isodesmic model, in which *K*_d_ (an initial dimerization process) is different to the *K* values for forming higher aggregates (Section S6b). Interestingly, the fit to the dimer and isodesmic models was poor (Figures S26 and S27), suggesting the aggregate formed by **1-***rac* is distinct to an H-type dimer^39^ or cofacial columnar stack,^44^ common assemblies for PDIs.^46^ Instead, the fact that spectral changes occur over a narrower concentration range indicates a nonisodesmic aggregation process (Figure 3a). Indeed, a “nucleation-elongation” mechanism is characteristic of slipped-stack J-type aggregates of PDIs.^66^ As such, the best fit was achieved with a cooperative nucleation-elongation model in which a weaker dimerization, *K*_d_ = 90 M^–1^, is followed by a stronger isodesmic extension of the aggregate, *K* = 90,000 M^–1^ (Figures 3a,b and S25).

It is notable that the H-type dimer formed by the bis-triazole PDI (*K*_d-inter_ = 1677 M^–1^ for **3**) is significantly stronger than the analogous J-type dimer (*K*_d_ = 90 M^–1^ for **1**). This is because, as evident from the crystal structure of macrocycle **1** (Figure 2c), the PDI’s bay triazole groups point away from the internal π-surfaces, thereby favoring a face-to-face H-type dimerization. The positioning of these heterocycles toward the outer π-surfaces of the dimer thus directs subsequent self-assembly through slipped-stack J-type π–π stacking (Figure 3b). This difference in *K*_d_ for H- and J-type aggregates is expected to be even larger in **1-***rac* since the macrocycle preorganizes the H-type dimer to make it *intra*molecular (so *K*_d-intra_ ≫ 1677 M^–1^), while the J-type dimer is intermolecular (*K*_d_ = 90 M^–1^).

Interestingly, this π–π self-assembly behavior is unique to the macrocycle racemate (**1-***rac*) since solutions of the pure enantiomers **1-MM***/***PP** continue to obey the Beer–Lambert law upon their concentration (Figure 3c), indicating the absence of higher-order π–π aggregates.^81^ Therefore, in stark contrast to *intra*molecular π–π stacking between PDIs, which is exclusively homochiral, the intermolecular π–π stacking of macrocycle **1** must be heterochiral. In other words, the studies with **1** indicate that, when both π-surfaces of a twisted 1,7-disubstituted PDI are available for π–π self-assembly, homochiral H-type dimerization on the less sterically hindered face is followed by heterochiral stacking on the remaining π-surface, which generates extended slipped-stack J-type aggregates (Figure 3b). While the J-type self-assembly of hydrogen-bonded tetrasubstituted PDIs have shown a dependence on optical purity,^62^ this is the first-time chirality has been connected to the simultaneous formation of H- and J-type aggregates of PDIs solely through complementary π–π interactions.

Distinct molecular packing in the single crystal structures of macrocycles **1-*****PP*** and **1-***rac* confirm the unique π–π self-assembled structure formed by the latter (Figure 3d,e). While both enantiopure and racemic crystals exhibit *intra*molecular H-type π–π stacking between the macrocycle’s two PDI units (*d* = 3.7 Å), only **1-***rac* exhibits intermolecular J-type π–π stacking (*d* = 4.0 Å, centroid–centroid slip-angle = 42°) between the PDI macrocycles themselves. Furthermore, this intermolecular π–π stacking in **1-***rac* is exclusively heterochiral, occurring between the ***M***-PDI unit of one macrocycle and the ***P***-PDI unit of a neighbor. Overall, the crystal packing shows how a mismatch in PDI chirality goes hand-in-hand with a slipped-stacked J-type arrangement, since the intermolecular π–π stacking can only occur between naphthalene subunits on adjacent macrocycles of opposite chirality.

These observations are consistent with the results of solution self-assembly studies. We confirmed that the self-assembled structure of **1-***rac* in solution matches that in the single crystal through agreement between solution and solid-state UV–vis spectra (Figures S20 and S24), most notably the presence of the same, red-shifted absorption band (λ = 593 nm), a feature not observed in crystals of **1-*****PP*** (Figure S20). Furthermore, the fluorescence emission in **1-***rac* crystals is red-shifted relative to that in the **1-*****PP*** crystals (Δλ = 23 nm, Figure S21). Therefore, macrocycle **1** is unique among twisted chiral PDI-based materials in exhibiting both H-type and J-type aggregation simultaneously.

### Chiroptical Properties

**Figure 4:**
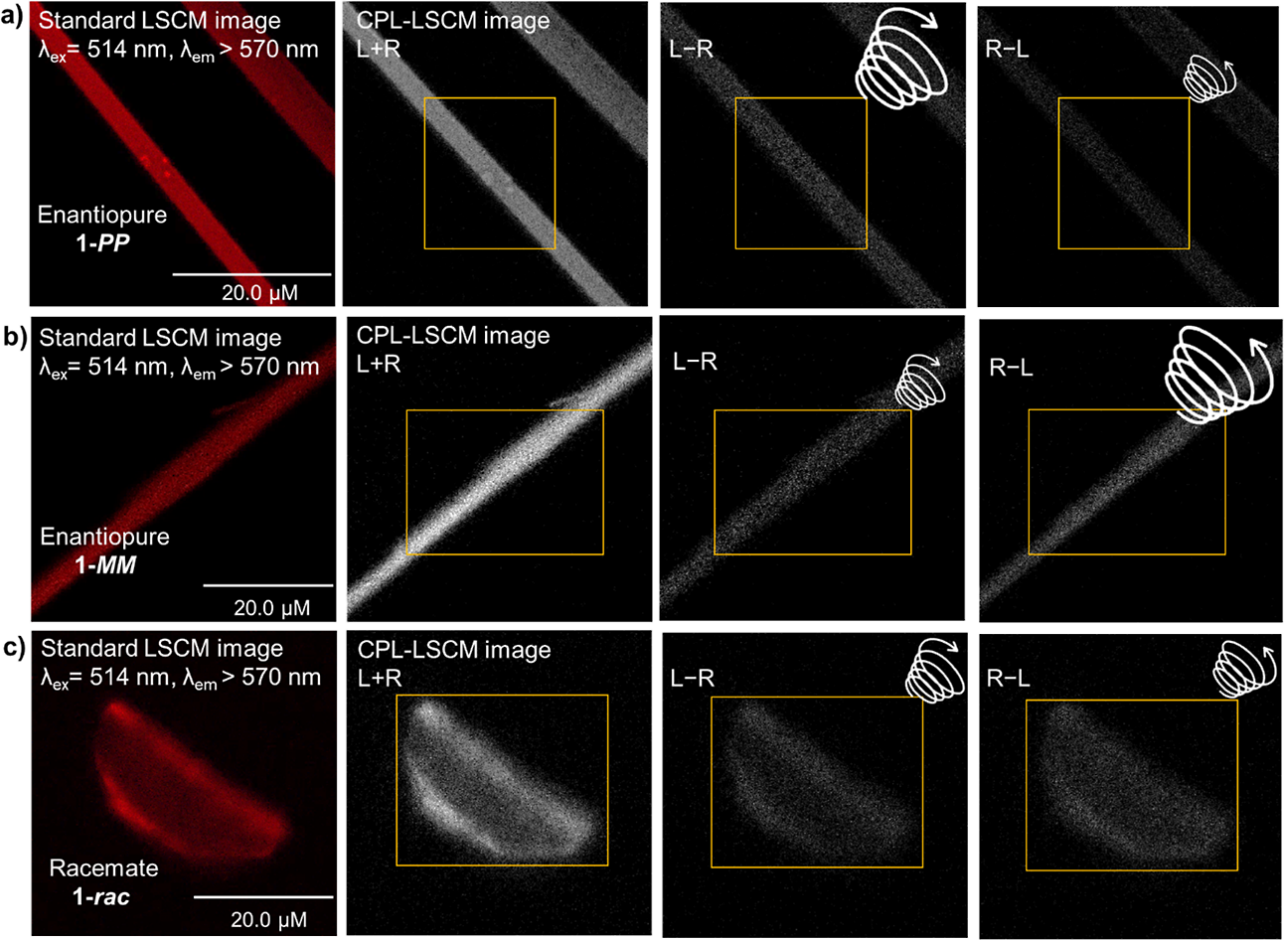
Standard and CPL-LSCM images (λ_em_ = 514 nm, Ar-laser, 2 mW) of single crystals of (a) **1***-***PP**, (b) **1***-***MM** and (c) **1***-rac*. The yellow squares denote the area where the image brightness was measured.

Having established the supramolecular organization of chiral macrocycle **1**, we sought to understand how the structure impacts chiroptical properties, including those in the solid state (Section S7). For this, we performed Enantioselective Differential Chiral Contrast (EDCC) imaging of single crystals of **1-***rac*, **1-*****PP***, and **1-*****MM*** using CPL Laser Scanning Confocal Microscopy (CPL-LSCM).^16^ In CPL-LSCM, right- and left-circularly polarized photons are collected simultaneously from the sample, generating independent right- and left-circular CPL images rapidly. To ascertain the difference between the degree of left-handed- and right-handed circularly polarized luminescence dominance, one of these images is subtracted from the other to generate an EDCC image. As such, we were able to perform EDCC imaging of **1-*****MM*** and **1-*****PP*** crystals because they emit equal and opposite CPL (Figures 4a,b, S32, and S33). Importantly, while the **1-***rac* and **1-MM***/***PP** crystals show emission in both the right- and left-handed CPL channels, only the enantiopure crystals exhibit a difference between the right and left CPL emission intensities (Figures 4a–c and S31–S33). These are the first reported CPL-LSCM EDCC images of single crystals.

**Figure 5:**
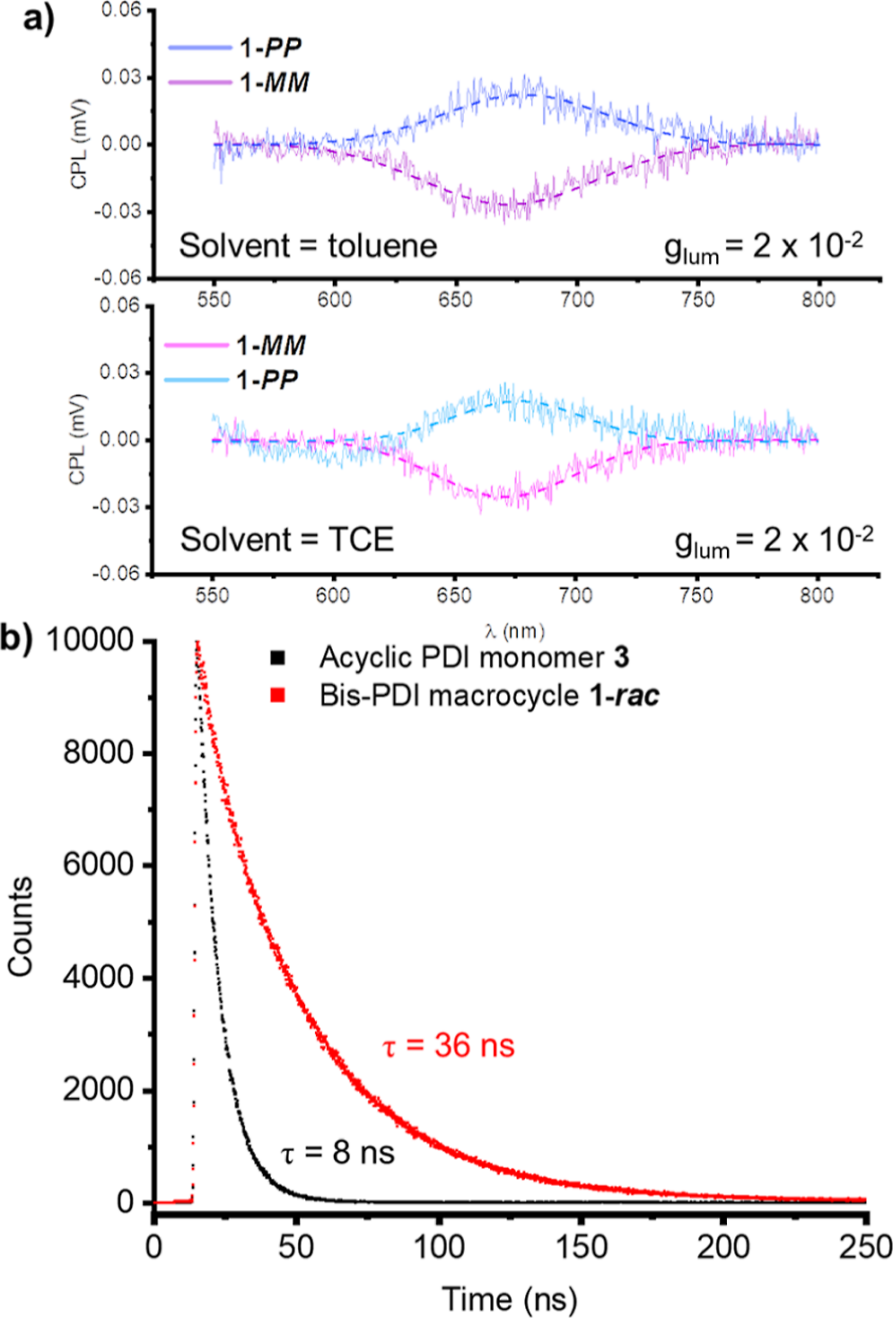
(a) Circularly polarized luminescence (CPL) spectra of **1-*****MM*** and **1-*****PP*** in toluene and TCE (10 μM, 298 K, λ_ex_ = 520 nm; dashed line is the Gaussian fit). (b) Time-resolved fluorescence decay profiles of **1-***rac* and PDI monomer **3** (toluene, 5 μmol, λ_ex_ = 373 nm, λ_em_ = 635 nm for **1-***rac*, and λ_em_ = 604 nm for **3**).

Having distinct enantiopure and racemic single crystals provided us with the opportunity to quantify the degree of circularly polarized emitted light from single crystals for the first time using CPL-LSCM, by calculating an EDCC dissymmetry factor (*g*_EDCC_), which is analogous to the luminescence dissymmetry factor obtained by CPL spectroscopy (*g*_lum_). In doing so, it is critical to correct for the inherent CPL bias arising from orientation-induced reflection and subsequent partial helicity inversion of emitted circularly polarized light. Therefore, we introduced a bias factor (*B* = 8 × 10^–3^), which is subtracted from the uncorrected (raw) EDCC values of the enantiopure crystals (Section S7c). Importantly, this bias factor could be easily determined by using the enantiopure **1-*****MM*** and **1-*****PP*** single crystal EDCC values. However, it is the **1-***rac* racemic crystals that enabled us to unequivocally determine and validate this system-specific bias factor as no overall CPL should be observed from **1-***rac* since it is a racemic material (Figure 4c). Incorporating the bias factor into eq 1, we then used (1) to calculate a *g*_EDCC_ of 6 × 10^–2^ at λ_em_ > 570 nm for **1-*****MM*** and **1-*****PP*** single crystals (Section S7c). We also performed CPL spectroscopy on **1-*****MM*** and **1-*****PP*** in toluene and TCE solution and calculated a *g*_lum_ = 2 × 10^–2^ at λ_em_ = 675 nm in both solvents (Figure 5a, Table 1). To put this in context, macrocycle **1** has a higher CPL dissymmetry factor than the majority of chirally locked small organic molecules in solution that emit in the red region of the spectrum (Figure S12).^10,28−30,33,82−87^
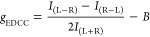
1where *g*_EDDC_ is the enantioselective differential chiral contrast (EDCC) dissymmetry factor, *I*_(L–R)_ is the left-handed EDCC average 8-bit (0–255 greyscale) pixel value^16^ (i.e., left CPL–right CPL), *I*_(L+R)_ is the right handed EDCC average 8-bit pixel value (i.e., right CPL–left CPL), *I*_(L+R)_ is the total image average 8-bit pixel value (left CPL + right CPL) and *B* is the bias factor (8 × 10^–3^).

Interestingly, the degree of emitted light circular polarization is three times higher in the crystalline state than in solution, most likely due to restricted molecular dynamics in the solid-state.^88−90^ This includes rigidification of the homochiral intramolecular π–π dimer, which, as found from self-assembly studies, is the primary PDI–PDI interaction in **1-*****MM***/***PP***. Indeed, we found that the strict homochirality of the intramolecular π–π dimer is key to the high emission dissymmetry factor of macrocycle **1**. This is because *g*_lum_ is an order of magnitude larger than the equivalent dissymmetry factor for absorption, *g*_abs_ = 3 × 10^–3^ (λ = 610 nm, Table 1), a characteristic feature of CPL emission from a chiral excimer state which arises from the excitonic coupling of two chromophores.^43,91,92^ Indeed, the fluorescence of macrocycle **1** is typical of excimer emission because, compared to the PDI monomer **3**, it is red-shifted (by 35 nm, Table 1 and Figures S13 and S15), quenched (Φ = 35% vs 70%, Table 1) and has a significantly longer lifetime (τ = 36 vs Eight ns, Figure 5b). Further evidence for the connection between homochiral π–π dimerization and *g*_lum_ comes from the fact that both are unchanged upon switching the solvent from toluene to TCE (Figures 5a, S13, and Table S1). Therefore, the π–π homochiral preorganization of chromophores, as evidenced from absorption and CD spectra, provides an effective strategy for amplifying *g*_lum_ in organic materials.

## Summary and Conclusions

We have designed and prepared a novel chiral bis-PDI macrocycle **1** that, due to our judicious choice of imide group, combines PDI configurational stability with accessible π-surfaces. These features enable us to develop a new understanding of the self-assembly of PDI chiroptical materials and their quantitative EDCC imaging using CPL-LSCM of single crystals for the first time. Configurational stability is bestowed by elongation of the macrocycle’s PDI units, which, as a novelty for bay-connected bis-PDI macrocycles, prevents the “intramolecular somersault” mechanism for stereoisomer interconversion.

Configurational stability enables us to assess the impact of disubstituted PDI chirality on the supramolecular structure and (chir)optical properties of self-assembled materials, both in solution and in the solid state. The π–π self-assembly of macrocycle **1** initially favors homochiral face-to-face (H-type) dimerization on the less sterically hindered interior π-surfaces (*K*_d_ ≫ 10^3^ M^–1^), followed by the formation of heterochiral slipped-stack (J-type) dimers (*K*_d_ ≈ 90 M^–1^), and ultimately extended J-type aggregates (*K* ≈ 10^4^ M^–1^), on the remaining exterior π-faces. Therefore, this allows us to make a more general prediction about the π–π self-assembly of 1,7-disubstituted PDIs: when both π-surfaces are available for π-stacking, homochiral H-type dimerization on the less sterically hindered face is then followed by heterochiral J-type slipped-stacking on the remaining π-surface.

As demonstrated by macrocycle **1**, this outcome has important consequences for the chiral structure–property relationships of 1,7-disubstituted PDI materials. First, the preference for homochiral H-type dimers leads to the diastereoselective synthesis of bis-PDI macrocycle enantiomers, **1-*****MM*** and **1-*****PP***, systems that are conducive to the formation of an intramolecular homochiral excimer to boost CPL. Notably, the degree of emitted light circular polarization is further amplified in single crystals of **1-MM***/***PP**, as shown by their enantioselective differential chiral contrast dissymmetry factor (*g*_EDCC_ = 6 × 10^–2^), a value akin to a *g*_lum_ and calculated here for the first-time using CPL-Laser Scanning Confocal Microscopy. Following dimerization, the self-assembly of 1,7-disubstituted PDI dimers requires a racemate and leads to red-shifted photophysics due to J-type aggregation. Therefore, we show that the chirality of the disubstituted PDI building block provides a rational route to designing H-, J-, and concomitant H- and J-type materials, with **1-***rac*, to the best of our knowledge, providing a unique demonstration of the latter for twisted PDIs. This discovery seeds the possibility of scalemic materials^59^ that synergize H- and J-type coupling between chiral chromophores to the mutual benefit of photon polarization and exciton transport, for the manipulation of circularly polarized light.

## Supporting Information Available

Experimental methods, including synthetic procedures, compound characterization, crystallographic and spectroscopic data, self-assembly studies, details and results of the (tight binding) DFT calculations, CPL microscopy, and computational studies (PDF)
Relevant DFT-optimized structures are available for download (ZIP)
