# Supplementary material for: A Chirally Locked Bis-perylene Diimide Macrocycle: Consequences for Chiral Self-Assembly and Circularly Polarized Luminescence
Source: J Am Chem Soc. 2024 Feb 14;146(8):5470–9. doi: 10.1021/jacs.3c13191 (PMC10910538; doi:10.1021/jacs.3c13191)
Supplement: Supplementary file 1 — ja3c13191_si_001.pdf [file ja3c13191_si_001.pdf]

A chirally-locked bis-perylene diimide macrocycle: consequences for chiral self-assembly and circularly polarized luminescence

Samuel E. Penty†, Georgia R. F. Orton†, Dominic Black‡, Robert Pal*,‡, Martijn A. Zwijnenburg§, Timothy A. Barendt*,†

†School of Chemistry, University of Birmingham, Edgbaston, Birmingham, B15 2TT, United Kingdom.

‡Department of Chemistry, University of Durham, South Road, Durham, DH1 3LE, United Kingdom.

§Department of Chemistry, University College London, 20 Gordon Street, London WC1H 0AJ, United Kingdom.

*t.a.barendt@bham.ac.uk

*robert.pal@durham.ac.uk

Supporting Information

Contents

1. Synthesis and Characterisation 2
2. HPLC analysis 13
3. X-ray crystallography 15
4. Chiroptical studies 19
5. Photophysics 22
6. Self-assembly studies 28
7. CPL microscopy 34
8. Computational studies 38
9. References 40

1. Synthesis and Characterisation

All commercial solvents and reagents were used as purchased, unless otherwise stated. Anhydrous solvents were degassed with N_2 and dried using an Innovative Technology PureSolv MD 5 solvent purification system. $Cu(MeCN)_4{\cdot}PF_6$ was stored in a desiccator. Tris((1-benzyl-4-triazolyl)methyl)amine (TBTA) was prepared following a literature procedure.[1] Water was distilled and microfiltered using an ELGA DV 35 Purelab water purification system. Chromatography was undertaken using silica gel (particle size: 40-63 μm) or preparative TLC plates (20 × 20 cm, 1 cm silica thickness).

^{1}H and ^{13}C NMR spectra were recorded using Bruker AVIII400 (400 MHz), Bruker AV NEO 400 (400 MHz) Bruker AV NEO 500 (500 MHz, with cryoprobe). Mass spectra were recorded using a Bruker UltrafleXtreme MALDI-TOF mass spectrometer or a Waters Synapt G2-S mass spectrometer for high resolution MS-ESI. Details of equipment used for other analytical techniques (CPL-LSCM microscopy, CD and CPL spectroscopies, photophysics, HPLC) are provided in their appropriate sections in this supporting information.

Synthesis of macrocycle 1

The synthesis of bis-PDI macrocycle **1** was carried out as shown in **Scheme S1**. Compound **4** was prepared according to a literature procedure[2] as a mixture of mono-, bis-, tris- and tetra-bromo isomers that, due to poor solubility, were separated at the compound **5** stage using silica gel column chromatography.

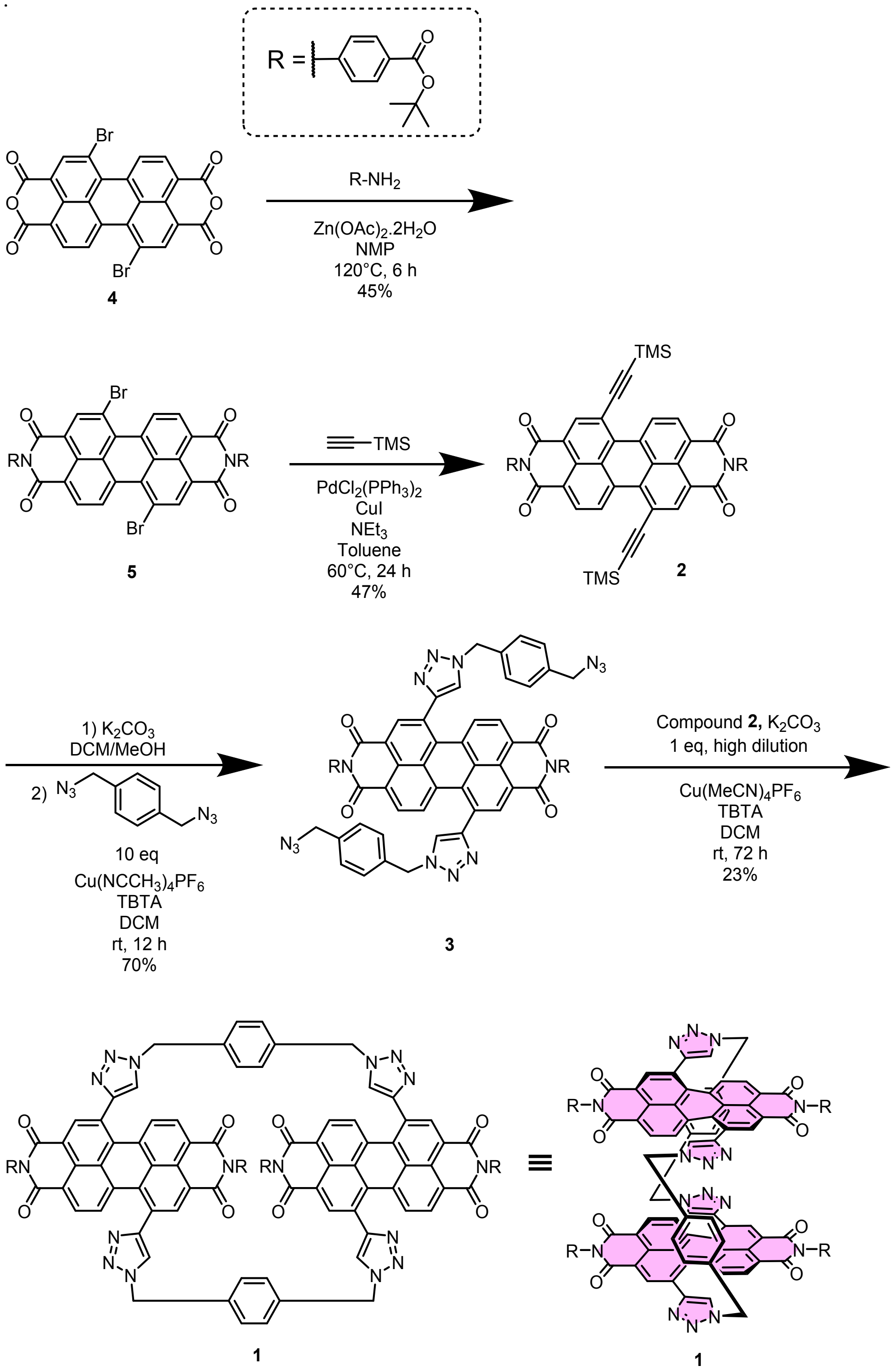

Scheme S1: Multistep synthesis of bis-PDI macrocycle **1**.

Bis-bromo PDI 5

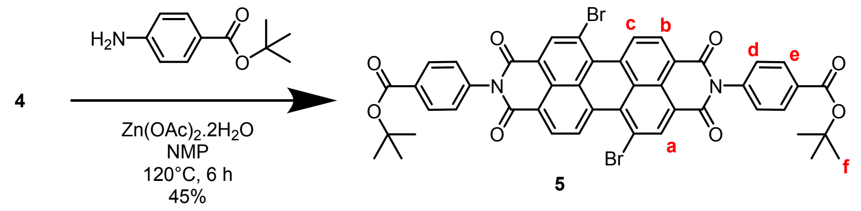

Compound **4** (a mixture of mono-, bis-, tris- and tetra-bromo perylenetetracarboxylic dianhydride, 1 g) was added to a flask containing N-Methyl-2-pyrrolidone (10 mL). To this was added tert-butyl 4-aminobenzoate (1.05 g, 5.45 mmol, 3 equiv) and $Zn(OAc)_2.2H_2O$ (678 mg, 3.09 mmol, 2 equiv). This mixture was purged with nitrogen and stirred at 120 °C for 6 hours. The reaction was then allowed to cool to rt, which yielded a red precipitate. The precipitate was collected by vacuum filtration, and thoroughly washed with water and then dried under vacuum. The resulting red residue was then purified by silica gel flash column chromatography (1:160 MeOH:$CHCl_3$), affording compound **5** as a mixture of 1,6 and 1,7 bis-bromo isomers (733 mg, 813 μmol, 45% yield if compound **4** was pure bis-bromo perylenetetracarboxylic dianhydride).

^{1}H NMR (500 MHz, Chloroform-*d*, 1,7-isomer) δ 9.58 (d, *J* = 8.1 Hz, $2H_b$), 9.01 (s, $2H_a$), 8.80 (d, *J* = 8.1 Hz, $2H_c$), 8.25 – 8.22 (m, $4H_d$), 7.44 – 7.42 (m, $4H_e$), 1.66 (s, $18H_f$).

^{13}C NMR (126 MHz, Chloroform-*d*) δ 164.9, 162.8, 162.3, 138.4, 138.3, 133.5, 133.3, 132.8, 130.7, 130.6, 129.5, 128.6, 127.4, 123.2, 122.8, 121.1, 81.5, 28.2.

HRMS (ESI) (m/z) calculated for $C_{46}H_{33}Br_2N_2O_8^+$ $[M+H]^+$ 899.0601, found 899.0604.

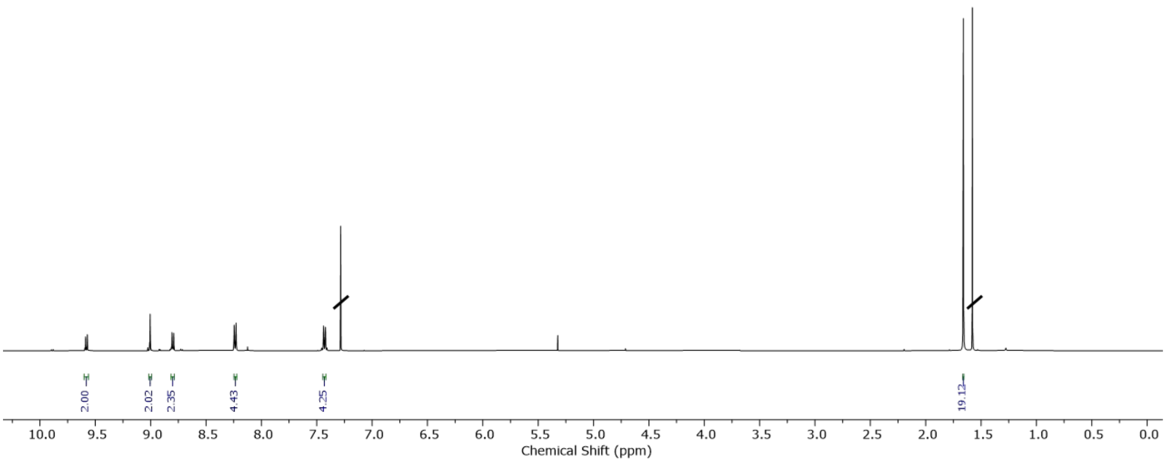

^{1}H NMR spectrum of compound **5** (chloroform-d, 298 K, 500 MHz)

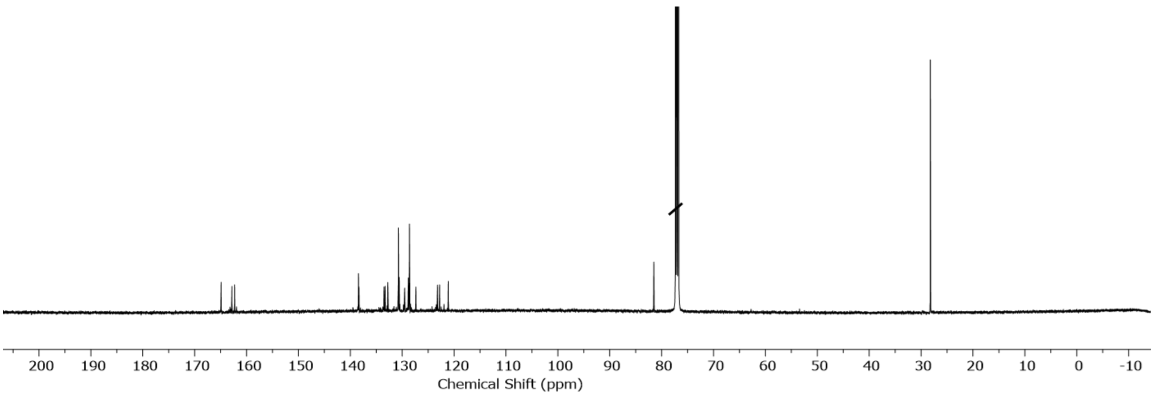

^{13}C NMR spectrum of compound **5** (chloroform-d, 298 K, 126 MHz)

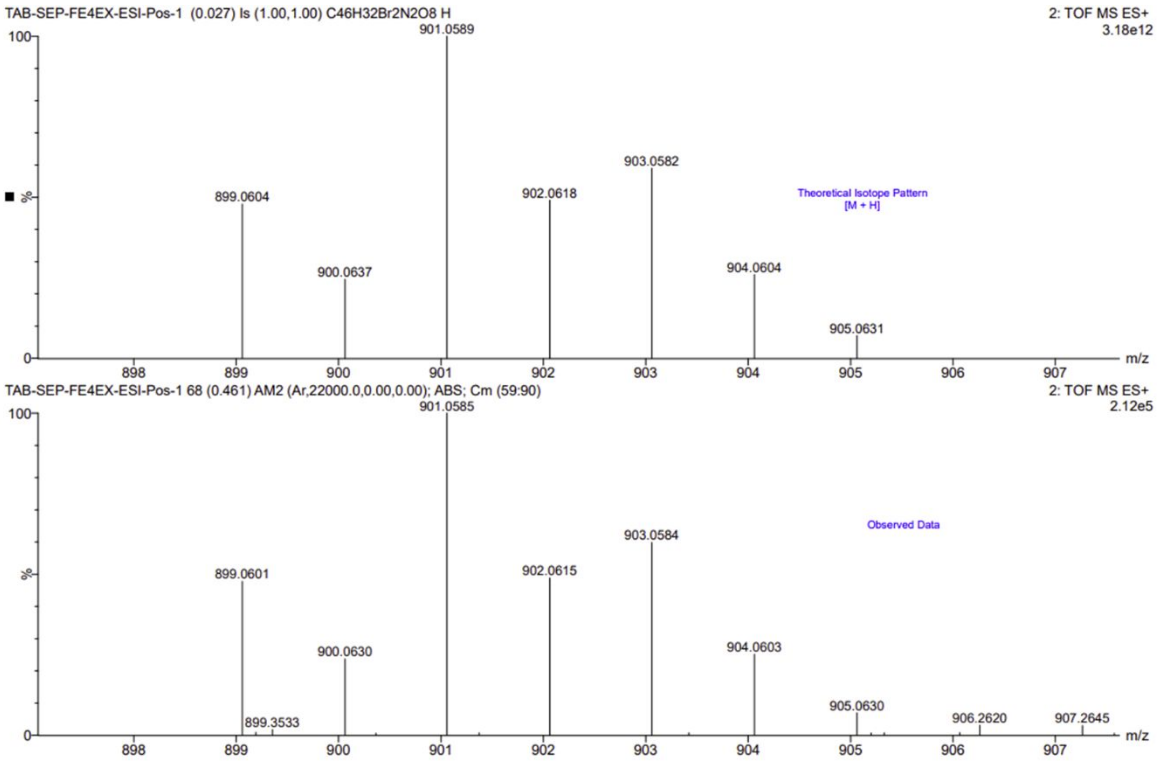

Calculated (top) and observed (bottom) ESI MS data for compound **5**.

TMS-protected bis-alkyne PDI 2

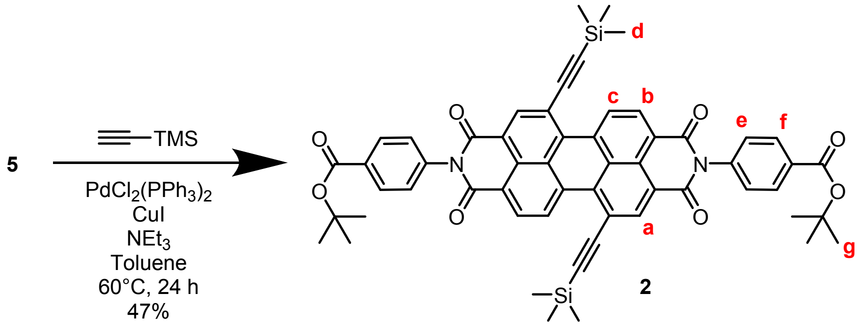

To a solution of bis-bromo PDI **5** (600 mg, 666 µmol) in 1:1 dry NEt_3-toluene (250mL) under a nitrogen atmosphere was added $Pd(PPh_3)_2Cl_2$ (28 mg, 40 µmol, 0.06 equiv), CuI (14 mg, 73 µmol, 0.11 equiv) and trimethylsilylacetylene (327 mg, 474 µL, 3.33 mmol, 5 equiv). The mixture was thoroughly de-gassed with nitrogen and stirred at 60 °C for 24 h. The solvent mixture was then removed in vacuo. The resulting residue was then re-dissolved in DCM (100 mL) and washed with water (3 x 50 mL); dried over anhydrous $MgSO_4$ and concentrated to dryness in vacuo. The resulting residue was purified by silica gel flash column chromatography (1:1 n-hexane:DCM) affording the title compound as a red solid (297 mg, 318 µmol, 48%).

^{1}H NMR (500 MHz, Chloroform-*d,* 1,7 isomer) δ 10.20 (d, *J* = 8.2 Hz, 2H_b), 8.83 (s, 2H_a), 8.65 (d, *J* = 18.5 Hz, 2H_c), 8.23 – 8.19 (m, 4H_e), 7.45 – 7.41 (m, 4H_f), 1.64 (s, 18H_g), 0.40 (s, 18H_d)

^{13}C NMR (126 MHz, Chloroform-*d*) δ 165.1, 163.2, 162.8, 138.9, 138.7, 138.5, 134.8, 134.5, 132.8, 131.1, 131.0, 130.8, 130.7, 129.0, 128.9, 128.8, 128.8, 128.1, 128.0, 127.7, 123.3, 122.2, 120.9, 120.4, 107.1, 106.8, 105.5, 105.5, 81.6, 29.9, 28.4, -0.2

HRMS (ESI) (m/z) calculated for $C_{56}H_{51}N_2O_8Si_2$ $[M+H]^+$ 935.3184, found 935.3182.

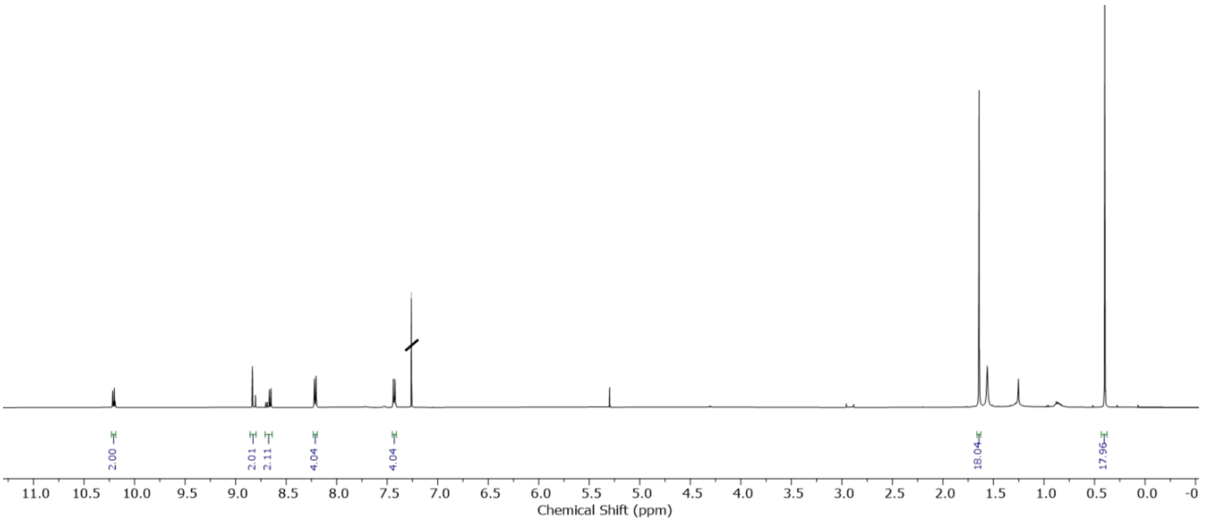

^{1}H NMR spectrum of compound **2** (chloroform-d, 298 K, 500 MHz)

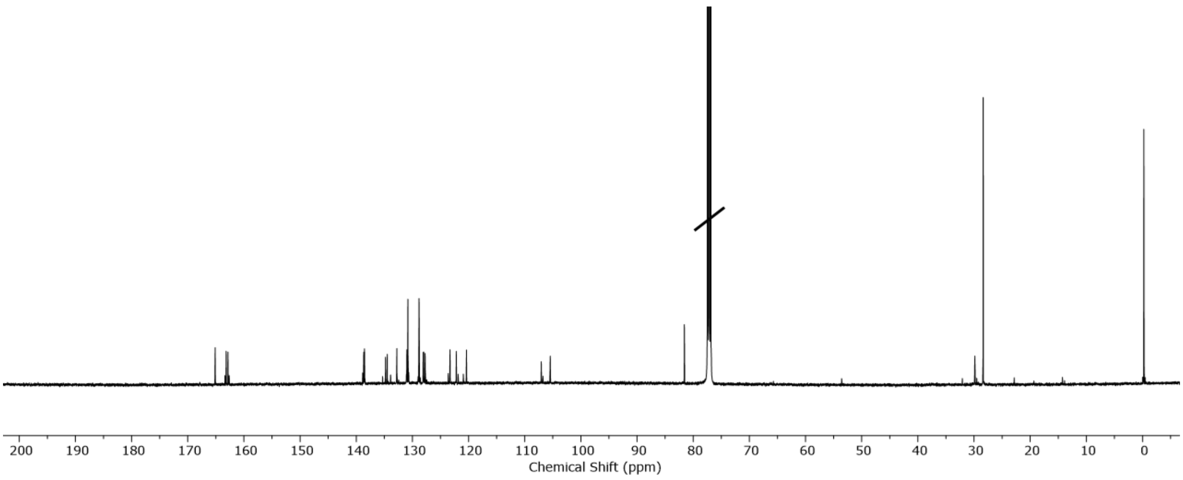

^{13}C NMR spectrum of compound **2** (chloroform-d, 298 K, 126 MHz)

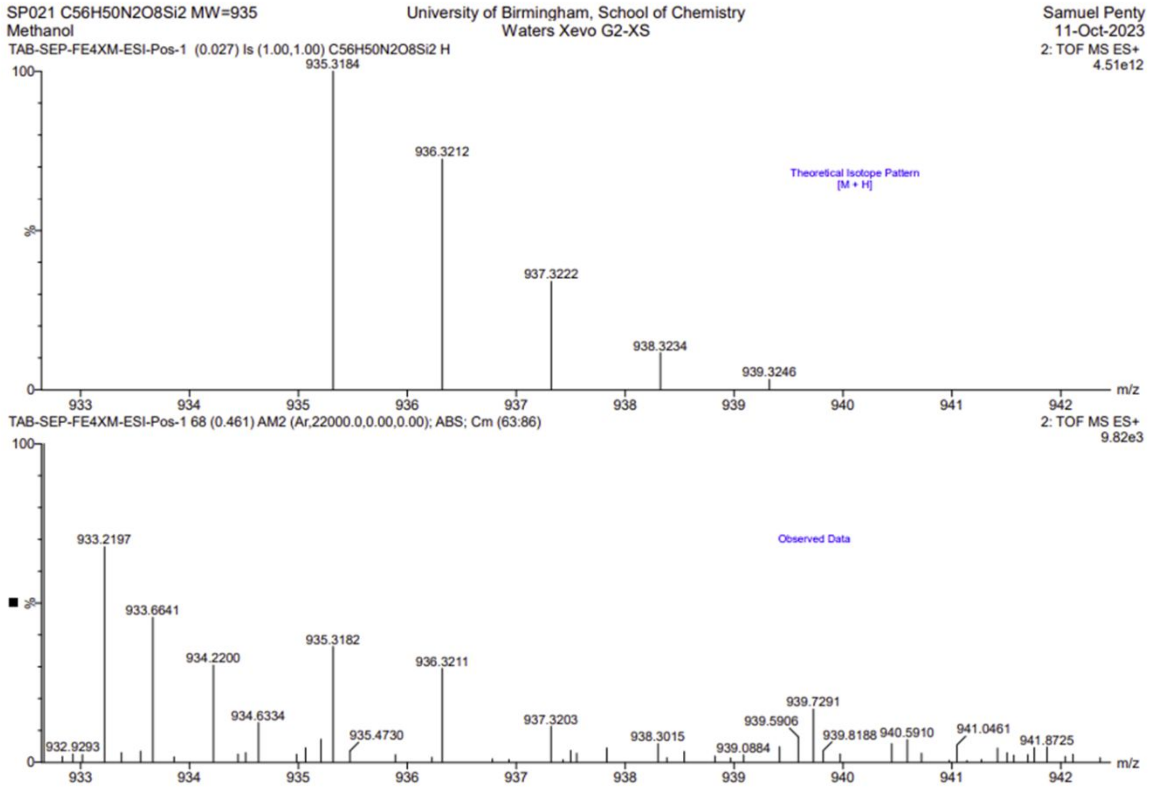

Calculated (top) and observed (bottom) ESI MS data for compound **2**.

Acyclic bis-triazole PDI 3

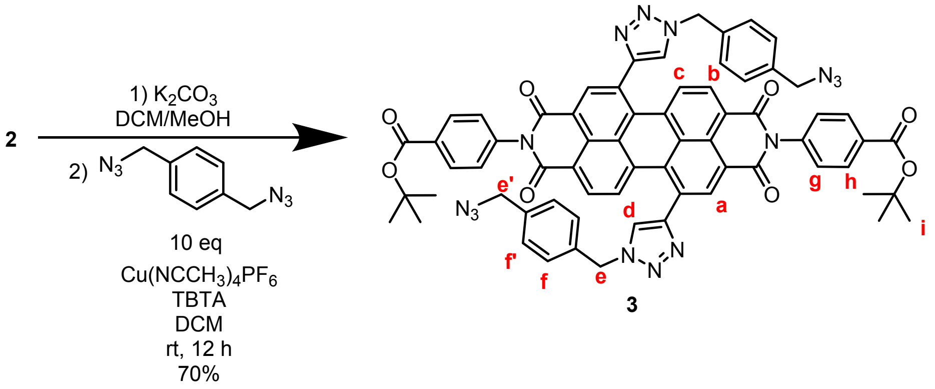

To a solution of TMS-protected bis-alkyne PDI **2** (70 mg, 75 µmol) in DCM (20 ml) was added K_2CO_3 (30 mg) in MeOH (10 ml). The mixture was stirred at rt for 3 min, and completion of the reaction was confirmed by TLC. The solution was then washed with water (2 x 30 mL) and brine (30 ml). The organic layer was then dried over anhydrous $MgSO_4$ and concentrated to dryness in vacuo to afford the deprotected PDI bis-alkyne which was used immediately without further purification. This PDI bis-alkyne was immediately re-dissolved in dry DCM (30 mL). To this was added 1,4- bis(azidomethyl)benzene (142 mg, 758 µmol, 10 equiv) and tris((1-benzyl-4- triazolyl)methyl)amine (TBTA) (16 mg, 30 µmol, 0.2 equiv). The solution was then de-gassed with argon. The copper (I) catalyst $Cu(CH_3CN)_4PF_6$ (11 mg, 30 µmol, 0.2 equiv) was then added and the solution was once again de-gassed with argon. The reaction was stirred at rt for 12 h. The solvent was then removed in vacuo. The resulting residue was purified by silica gel flash column chromatography (1:99 MeOH-DCM) affording the title compound as a purple solid (62 mg, 53 µmol, 70%).

^{1}H NMR (500 MHz, Chloroform-*d,* 1,7 isomer) δ 8.67 (s, $2H_a$), 8.22 – 8.17 (m, $4H_g$), 8.15 (d, *J* = 8.1 Hz, $2H_b$), 7.84 (d, *J* = 8.0 Hz, $2H_c$), 7.63 (s, $2H_d$), 7.40 – 7.35 (m, $12H_{h,f,f'}$), 5.64 (s, $4H_e$), 4.36 (s, $4H_{e'}$), 1.63 (s, $18H_i$).

^{13}C NMR (126 MHz, Chloroform-*d*) δ 165.1, 163.2, 163.1, 148.1, 138.8, 136.9, 135.3, 135.0, 134.6, 134.0, 132.7, 130.8, 130.2, 130.0, 129.5, 129.4, 129.3, 129.2, 129.1, 128.8, 128.7, 128.6, 128.6, 122.7, 122.5, 122.1, 121.9, 81.6, 54.4, 54.3, 28.4.

HRMS (ESI) (m/z) calculated for $C_{66}H_{50}N_{14}O_8^+$ $[M+H]^+$ 1167.4015, found 1167.4026.

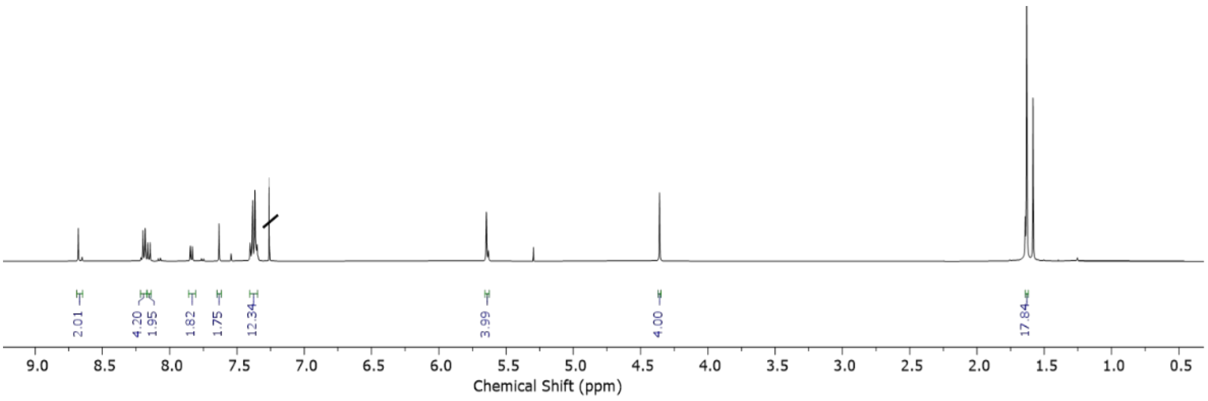

^{1}H NMR spectrum of compound **3** (chloroform-*d*, 298 K, 500 MHz)

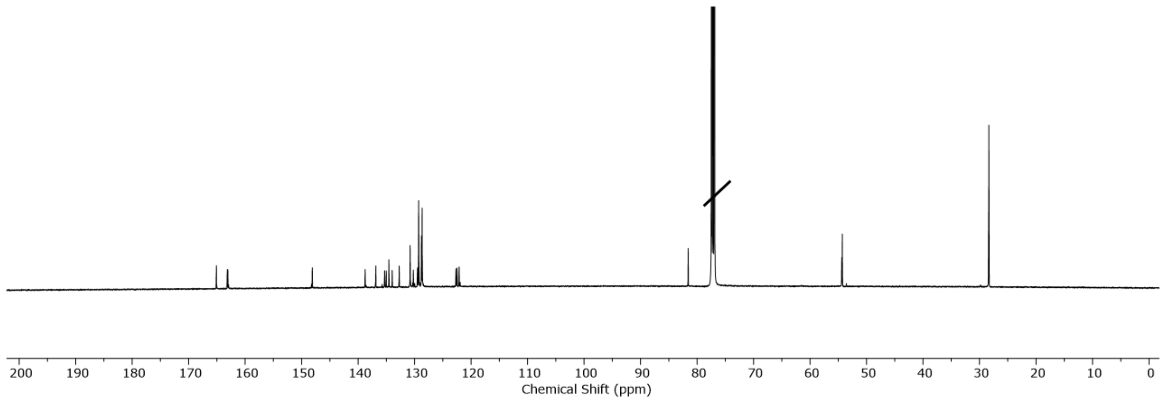

^{13}C NMR spectrum of compound **3** (chloroform-*d*, 298 K, 126 MHz)

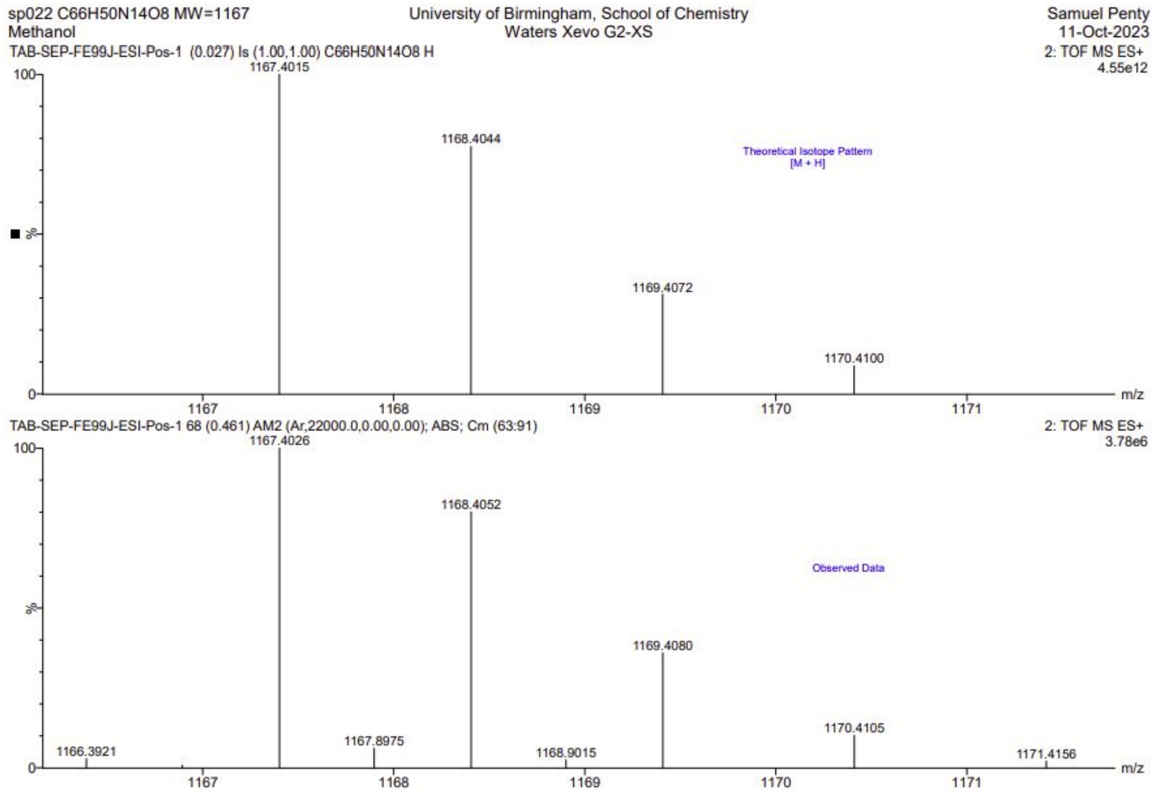

Calculated (top) and observed (bottom) ESI MS data for compound **3**.

Bis-PDI macrocycle 1

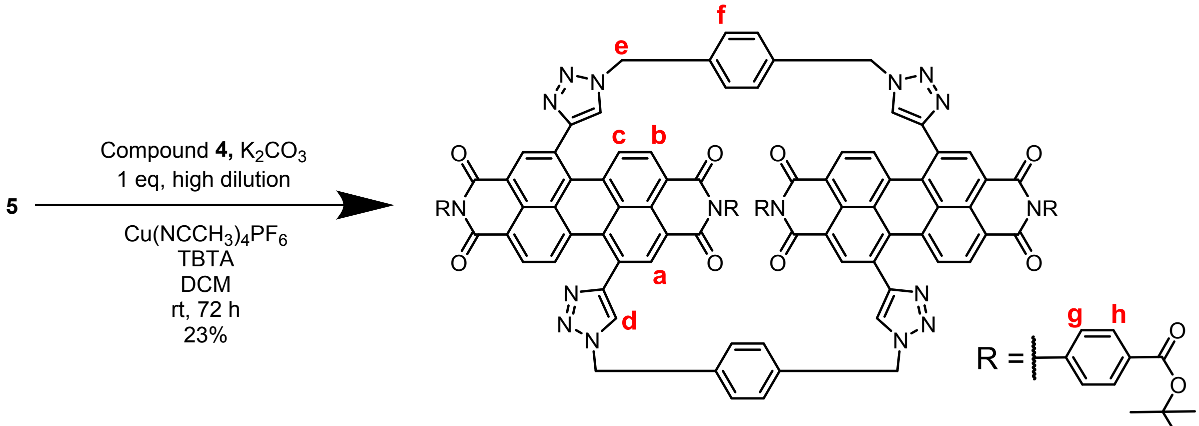

TMS-protected bis-alkyne PDI **4** (50 mg, 53 µmol) was dissolved in DCM (20 mL). To this was added K_2CO_3 (20 mg) in MeOH (10 mL). The reaction was monitored by TLC (1:99 MeOH:DCM). Upon completion the reaction mixture was thoroughly washed with water in a separating funnel (3 x 100 mL) and dried with $MgSO_4$ to yield crude deprotected bis-alkyne PDI in DCM, which was used immediately without further purification due to its tendency to aggregate and crash out of solution over time. This was added to a flask, along with acyclic bis-triazole PDI **5** (62 mg, 53 µmol, 1 equiv), tris((1-benzyl-4-triazolyl)methyl)amine (TBTA) (12 mg, 21 µmol, 0.4 eq) and a further 350 mL of DCM. The reaction mixture was thoroughly de-gassed with N_2. The copper catalyst $Cu(CH_3CN)_4PF_6$ (8 mg, 21 µmol, 0.4 equiv) was then added and the reaction mixture was thoroughly de-gassed again. The reaction was stirred at rt for 36 h and monitored by TLC (2:98 MeOH-DCM). The solvent was then removed *in vacuo*. The resulting residue was dissolved in DCM and filtered through cotton wool and the filtrate was then purified by preparative silica TLC (2:98 MeOH-DCM), affording the title compound (as the pure 1,7-regioisomer) as a purple solid (24 mg, 12 µmol, 23%).

^{1}H NMR (400 MHz, 373K, TCE-*d*$_2$) δ 8.28 (s, 4H$_d$), 8.23 (d, J = 8.2 Hz, 8H$_g$), 8.20 (s, 4H$_a$), 7.96 (s, 8H$_f$), 7.64 (d, J = 8.3 Hz, 4H$_b$), 7.58 (d, J = 8.2 Hz, 8H$_h$), 6.05 (d, J = 13.8 Hz, 4H$_e$), 5.64 (d, J = 8.3 Hz, 4H$_c$), 5.59 (d, J = 13.9 Hz, 4H$_e$), 1.72 (s, 36H$_i$).

^{13}C NMR (101 MHz, TCE-*d*$_2$) δ 165.1, 162.0, 138.4, 133.4, 130.7, 130.1, 129.4, 129.2, 121.5, 99.9, 81.9, 74.5, 74.2, 73.9, 32.0, 29.8, 29.4, 29.2, 28.5, 25.0, 22.8, 14.1.

HRMS (ESI) (m/z) calculated for $C_{116}H_{85}N_{16}O_{16}^+$ [M+H]$^+$ 1957.6329, found 1957.6272

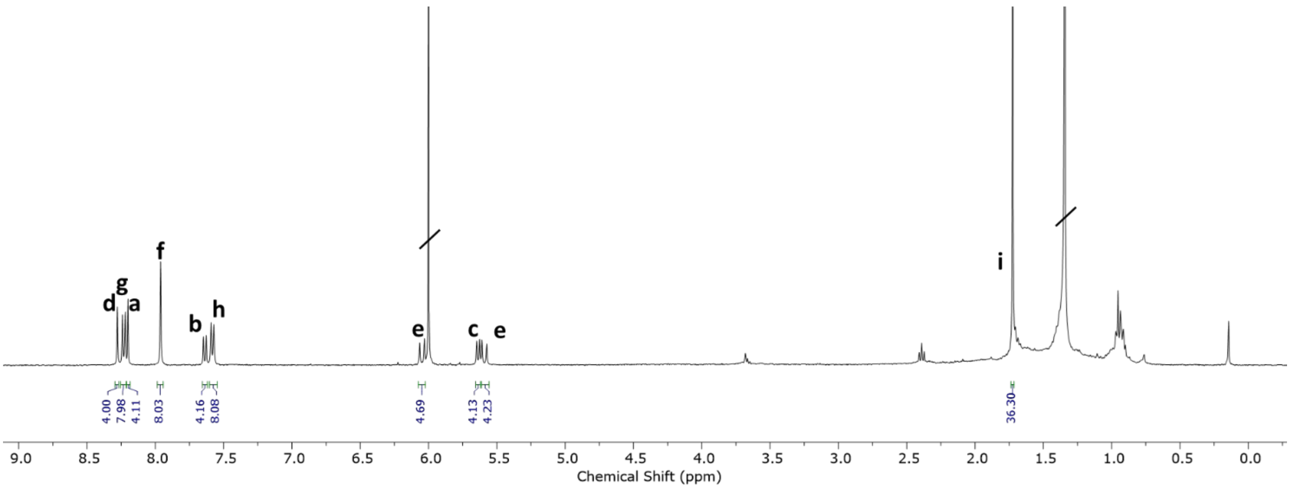

^{1}H NMR spectrum of macrocycle **1** (TCE-d_2, 373 K, 400 MHz)

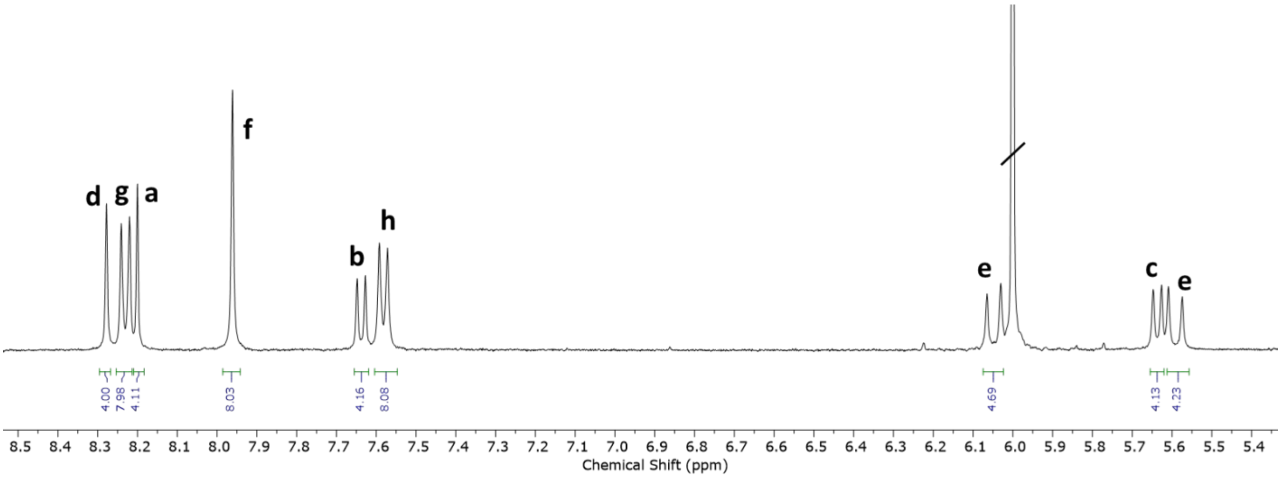

Aromatic region of the ^{1}H NMR spectrum of macrocycle **1** (TCE-d_2, 373 K, 400 MHz)

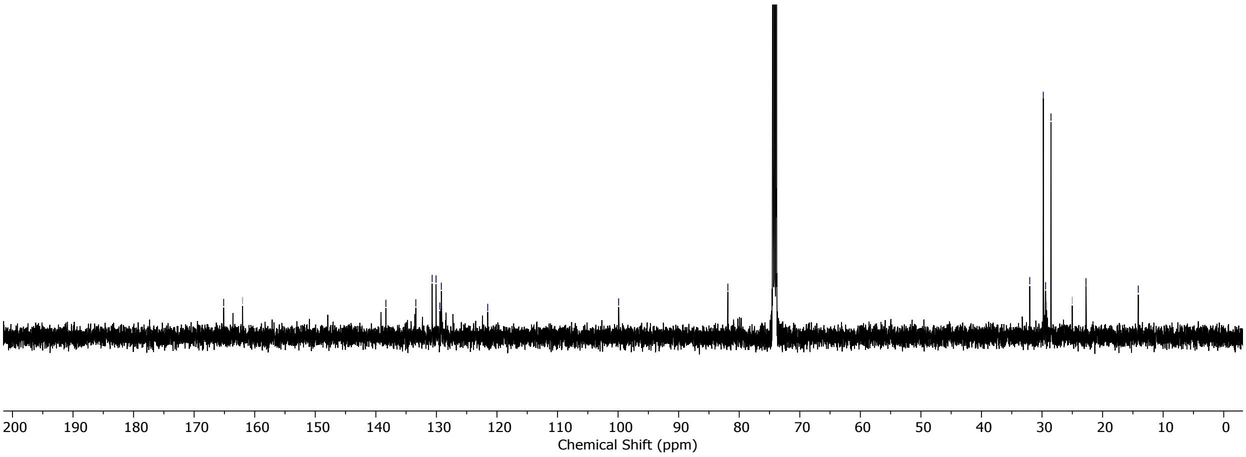

^{13}C NMR spectrum of macrocycle **1** (TCE-d_2, 373 K, 400 MHz). The poor signal to noise ratio is due to the large number of quaternary carbons and the somewhat low solubility of macrocycle **1**.

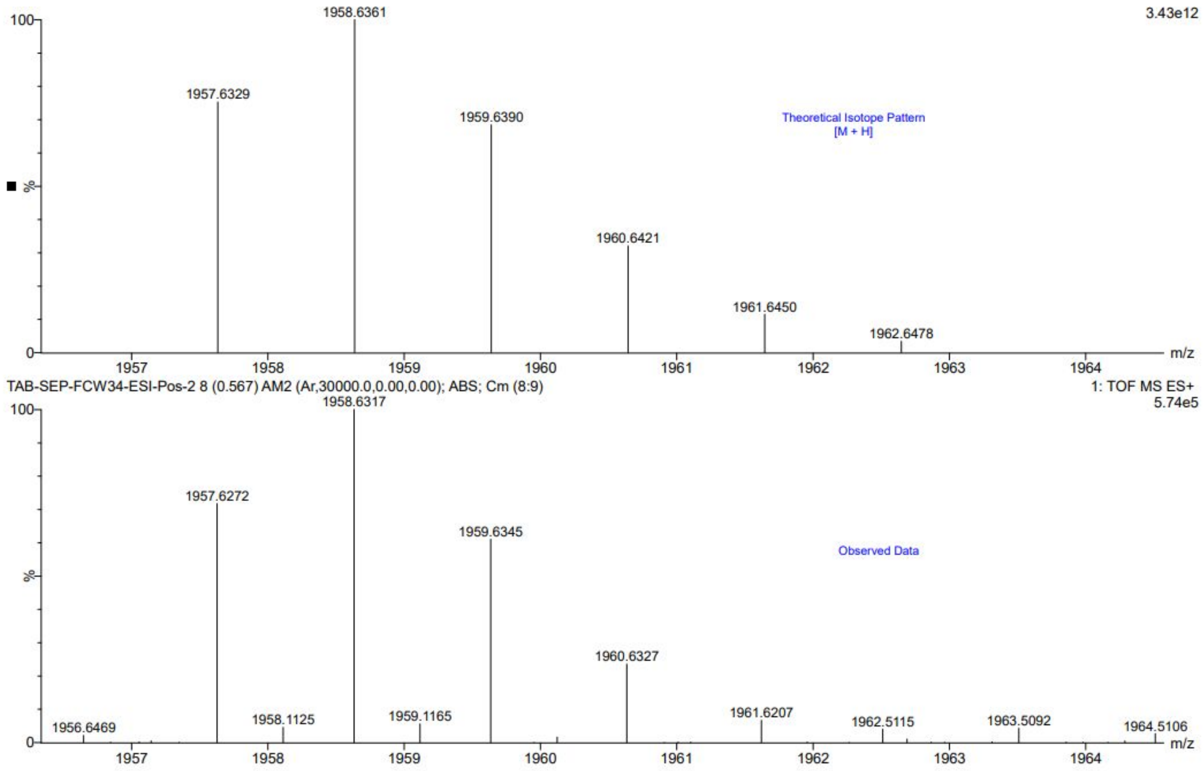

Calculated (top) and observed (bottom) ESI MS data for macrocycle **1**.

2. HPLC analysis

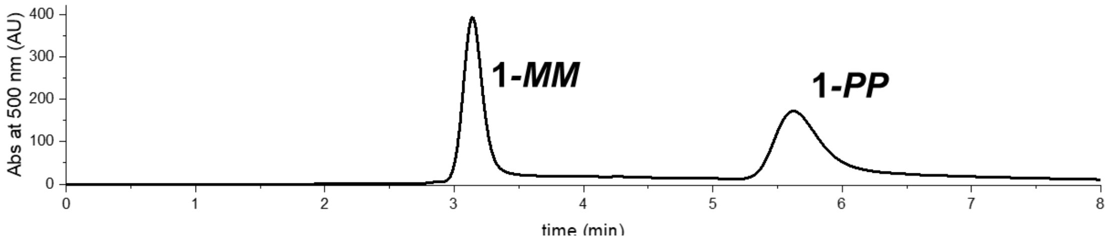

Figure S1: Chiral HPLC chromatogram (Phenomenex i-Amylose-1, 250 x 4.6 mm) of **1-*rac*** dissolved in DCM and eluted with 9:1 (v/v) DCM:methanol. The peaks were assigned as the ***MM*** and ***PP*** enantiomers by single crystal X-ray crystallography of the enantiopure crystals of ***1-PP*** (the second peak). The two peaks have equal integral areas.

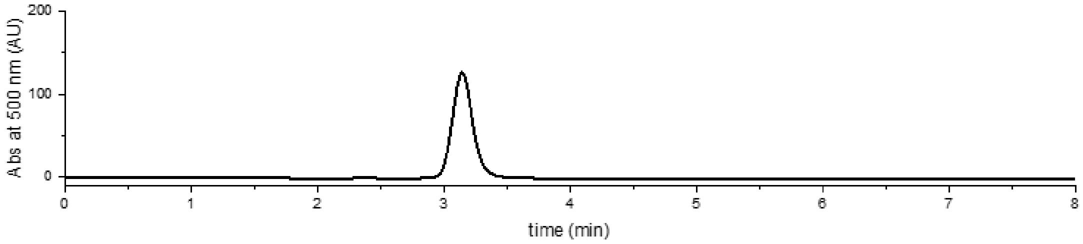

Figure S2: Chiral HPLC chromatogram (Phenomenex i-Amylose-1, 250 x 4.6 mm) of a sample of enantiopure **1-*MM***, previously purified by chiral HPLC and immediately reinjected into the HPLC column. Eluted with 9:1 (v/v) DCM:methanol.

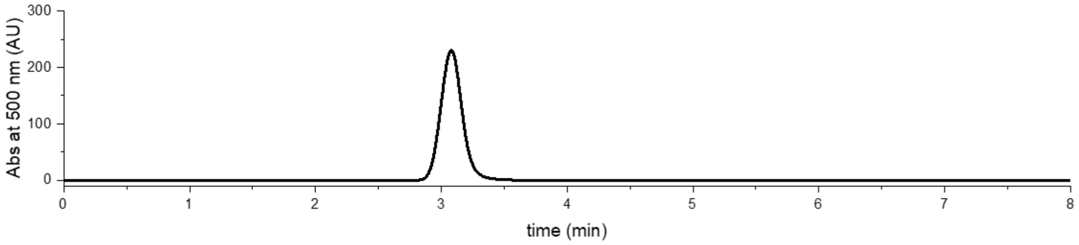

Figure S3: Chiral HPLC chromatogram (Phenomenex i-Amylose-1, 250 x 4.6 mm) of a sample of enantiopure **1-*MM***, previously purified by chiral HPLC, that was heated at 180 °C for 24 h in 1,2-dicholorbenzene. After heating, the solvent was removed and the sample was re-dissolved in DCM and reinjected into the chiral HPLC column and eluted with 9:1 (v/v) DCM:methanol. No racemisation to form the other enantiomer **1-*PP*** can be detected in this chromatogram, proving that macrocycle **1** is chirally locked at temperatures at least as high as 180 °C.

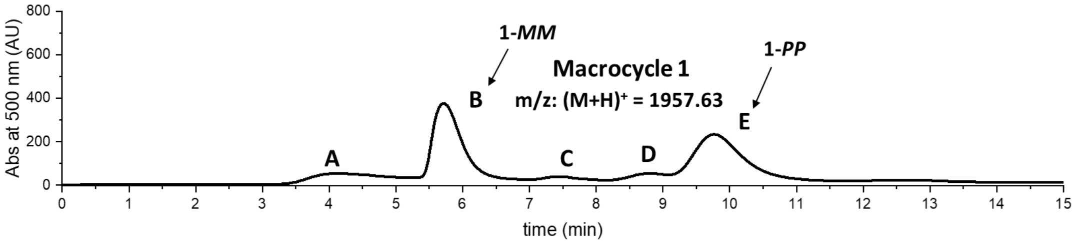

Figure S4: Chiral HPLC chromatogram (Phenomenex i-Amylose-1, 250 x 10 mm) of the crude reaction mixture of macrocycle **1**. The sample was microfiltered prior to injection into the HPLC column to remove insoluble oligomeric side-products, but no other purification was carried out prior to injection. Peaks A-E were collected and analysed by ESI-MS. Macrocycle **1** was only present in peaks B and E (m/z = 1958) which, as identified by CD-spectroscopy and assigned by TD-DFT, are **1-*MM*** and **1-*PP*** respectively.

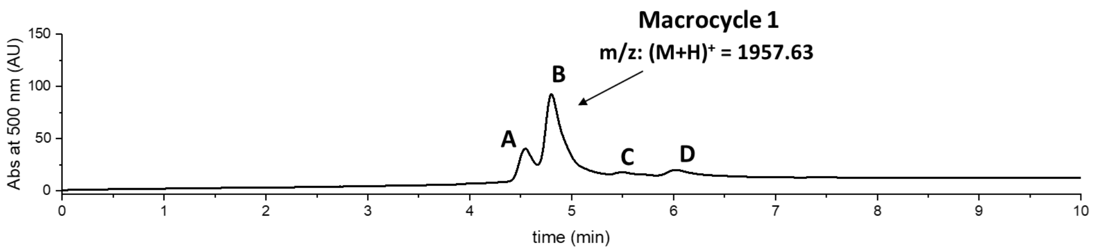

Figure S5: HPLC chromatogram (COSMOSIL Buckyprep 250 x 10 mm) of the crude reaction mixture of macrocycle **1**. The sample was microfiltered prior to injection into the HPLC column to remove insoluble oligomeric side-products, but no other purification was carried out prior to injection. Peaks A-D were collected and analysed by ESI-MS. Macrocycle **1** was only present in peak B (m/z = 1958) which, when resolved by chiral HPLC (Figure S1), contains only the enantiomers **1-*MM*** and **1-*PP***.

3. X-ray crystallography

a) Macrocycle 1-*rac*

Purple, needle-like crystals of macrocycle **1-*rac***, suitable for single crystal X-ray diffraction, were grown by slow diffusion of methanol into a chloroform solution.

Single crystal X-ray diffraction experiments were performed by the UK National Crystallography Service on a Rigaku 007HF diffractometer with HF Varimax confocal mirrors, an UG2 goniometer and HyPix 6000HE detector.

The crystals were kept at 100(2) K during data collection. The structures were solved by direct methods using ShelXT[3] and refined with ShelXL[4] using a least squares method. Olex2 software was used as the solution, refinement and analysis program.[5]

The crystal diffracted weakly with a low-resolution diffraction limit; the data was truncated to a resolution of 1.00 A. Many attempts were made to grow stronger diffracting crystals, and this sample was sent to the synchrotron on several occasions. This dataset from the UK National Crystallography Service (NCS) gave the best refinement.

All non-hydrogen atoms were refined anisotropically. All hydrogen atoms were geometrically placed and refined using a riding model. Owing to the weak data, all methyl groups were placed in eclipsed conformations (AFIX 33) rather than having their torsion angles refined against a search of the Fourier map. This method aided convergence of the refinement. Restraints and constraints were applied to the structure to aid refinement. The anisotropic displacement parameters of tertiary carbon atoms C32, C43, C78, and C89 in the t-butyl ester groups were constrained to be identical (EADP). The anisotropic displacement parameters of carbon atoms C90, C91, and C92, which from a t-butyl group with tertiary carbon C86, were constrained to be identical (EADP) and the C-C bond lengths of the bonds carbon atoms C90, C91, C92 form with carbon atom C89 were restrained to have similar distances (SADI). The anisotropic displacement parameters of CH3 carbon atoms C35 C34 C33 C45 C44 C46 C79 C80 C81 of the remaining three t-butyl groups were restrained to be similar (SIMU). The anisotropic displacement parameters of oxygen atoms O1, O2, O3, O4, O9, O10, O11, and O12 were restrained to be similar (SIMU).

Due to poor data quality residual solvent could not be sensibly modelled. A solvent mask was calculated and 469 electrons were found in a volume of 1757 cubic angstrom in 1 void per unit cell. This is consistent with the presence of 13 methanol molecules per Asymmetric Unit which account for 468 electrons per unit cell.

Crystal Data for macrocycle **1-*rac***: $C_{116}H_{84}N_{16}O_{16}$ (M = 1958 g/mol): triclinic, space group P-1, a = 17.357(3) Å, b = 18.793(3) Å, c = 21.113(3) Å, α = 103.153(12)°, β = 94.994(11)°, γ = 115.198(16)°, V = 5934.2(17) Å^3, Z = 2, T = 100.15 K, μ(Cu Kα) = 0.784 mm^{-1}, *Dcalc* = 1.329 g/cm^3, 30226 reflections measured (5.436° ≤ 2Θ ≤ 100.868°), 11839 unique (R_{int} = 0.1886, R_{sigma} = 0.3929) which were used in all calculations. The final R_1 was 0.1226 (I > 2σ(I)) and wR_2 was 0.4448 (all data). Deposited cif number: 2308898

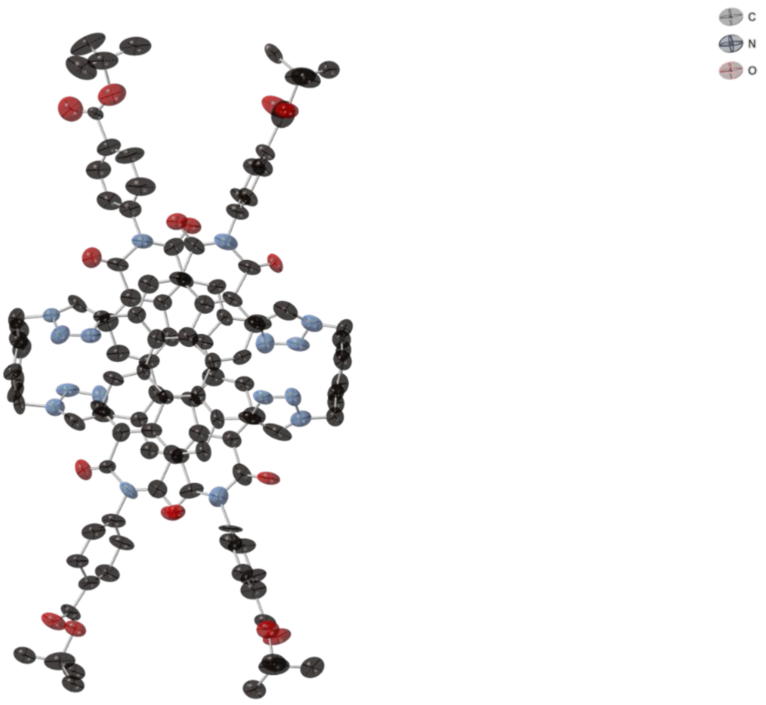

Figure S6: Molecular unit of macrocycle **1** from the **1-*rac*** crystal structure b with all non-hydrogen atoms represented by ellipsoids at the 25% probability level. Hydrogen atoms omitted for clarity (C, black; O, red; N, blue).

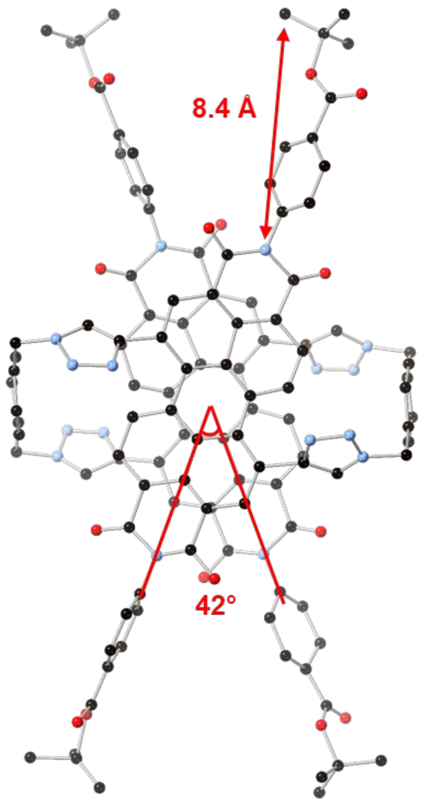

Figure S7: Crystal structure of macrocycle **1** from the **1-*rac*** crystal structure depicting the length of the imide groups and the rotational displacement of the PDI cores.

b) Enantiopure macrocycle 1-*PP*

Needle-like crystals of enantiopure macrocycle **1-*PP*** suitable for single crystal X-ray diffraction were grown from an enantiopure sample of **1-*PP*** by slow diffusion of hexane into a 1:1 chloroform:1,2-dicholobenzene solution.

Single crystal X-ray diffraction experiments were performed at the UK Diamond Light Source I19-1 3-circle diffractometer (λ = 0.6889 Å).[6-8] A suitable single crystal was selected and mounted using fomblin film on a micromount. Data were collected on a dectris-CrysAlisProabstract goniometer imported dectris images diffractometer. The crystals were kept at 100(2) K during data collection. The structures were solved by direct methods using ShelXT,[3] and refined with ShelXL[4] using a least squares method. Olex2 software was used as the solution, refinement and analysis program.[5] Figures were produced using CrystalMakerX.

All non-hydrogen atoms were refined anisotropically. Positional disorder is modelled for carbon atoms C44A and C44B, C45A and C45B, C46A and C46B. The occupancies of the two sites were refined and constrained to sum to unity. Their occupancies refined to 0.5 so their occupancies were then set to 0.5. The anisotropic displacement parameters of carbon atoms C44A, C44B, C45A, C45B, C46A, and C46B were constrained to be similar (**SIMU**). The C-C bond lengths of the C43 with C44, C45, and C46 were restrained to be the same (**EADP**).

All hydrogen atoms were geometrically placed and refined using a riding model. Methyl hydrogens on carbon atoms C44A, C44B, C45A, C45B, C46A, and C46B were placed in eclipsed conformations (**AFIX 33**) rather than having their torsion angles refined against a search of the Fourier map. This method aided convergence of the refinement.

The majority of disordered solvent molecules could not be sensibly modelled. Carbon atom C117 is the pivot point (occupancy 1) for several possible orientations of one disordered chloroform model. Chlorine atoms Cl1, Cl2, and Cl3 are modelled with occupancy 0.5. Although further Cl orientations for this disordered molecule could be observed in the electron density map, they could not be sensibly modelled. A solvent mask was calculated using the Olex implementation of SQUEEZE and 394 electrons were found in a volume of 1218 cubic angstroms in 1 void per unit cell. This is consistent with the presence of 3.4[CCl3H] per Asymmetric Unit which account for 394 electrons per unit cell.

Crystal Data for : $C_{116}H_{84}N_{16}O_{16}$ (M = 1958 g/mol): monoclinic, space group $P2_1$ (no. 4), a = 12.2125(4) Å, b = 20.4026(4) Å, c = 23.5546(5) Å, β = 104.554(2)°, V = 5680.7(3) Å^3, Z = 2, T = 100 K, μ(Synchrotron) = 0.332 mm^{-1}, $Dcalc$ = 1.421 g/cm^3, 96354 reflections measured (2.596° ≤ 2Θ ≤ 51.006°), 21937 unique (R_{int} = 0.0769, R_{sigma} = 0.0806) which were used in all calculations. The final R_1 was 0.0659 ($I > 2\sigma(I)$) and wR_2 was 0.1938 (all data). Deposited cif number: 2308894

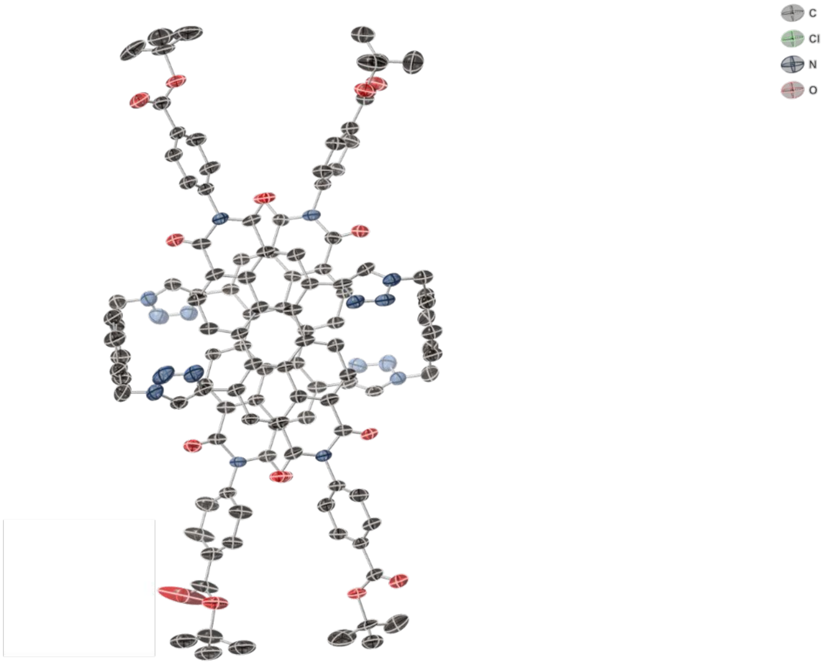

Figure S8: Molecular unit of macrocycle **1** from the **1-*PP*** crystal structure with all non-hydrogen atoms represented by ellipsoids at the 50% probability level. Hydrogen atoms omitted for clarity (C, black; O, red; N, blue).

4. Chiroptical studies

a) Circular dichroism

Circular dichroism (CD) spectra were recorded on a Jasco J-1500 CD spectrophotometer with a wavelength accuracy ± 0.2 nm (250 to 500 nm), ± 0.5 nm (500 to 800 nm) and a CD root mean square noise < 0.007 mdeg (500 nm). A quartz cuvette with 1 mm path length was used. The spectra were recorded at a concentration of 10 μM. The enantiomers were assigned by corroboration of the samples with the crystal structure of enantiopure **1-*PP***, as well as by comparison of their CD spectra to the TD-DFT computationally predicted CD spectra (Table S4).

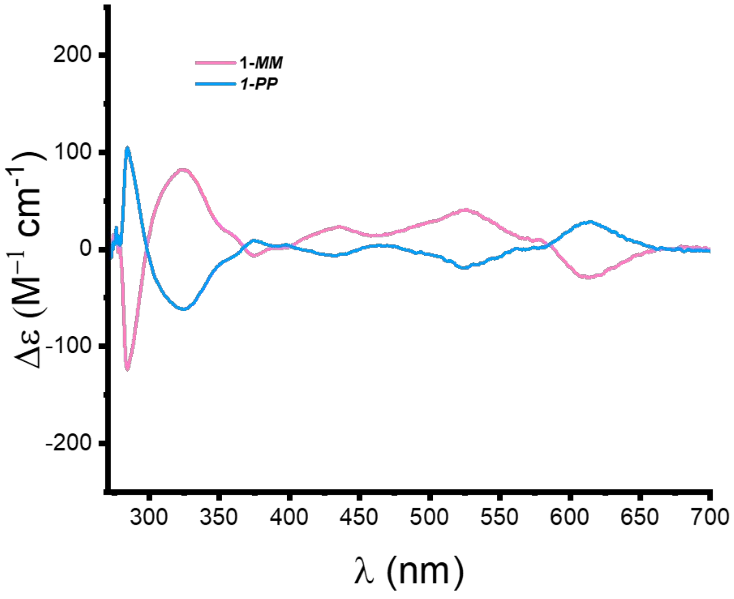

Figure S9: CD spectra for the **1-*MM*** and **1-*PP*** enantiomers of macrocycle **1** in toluene.

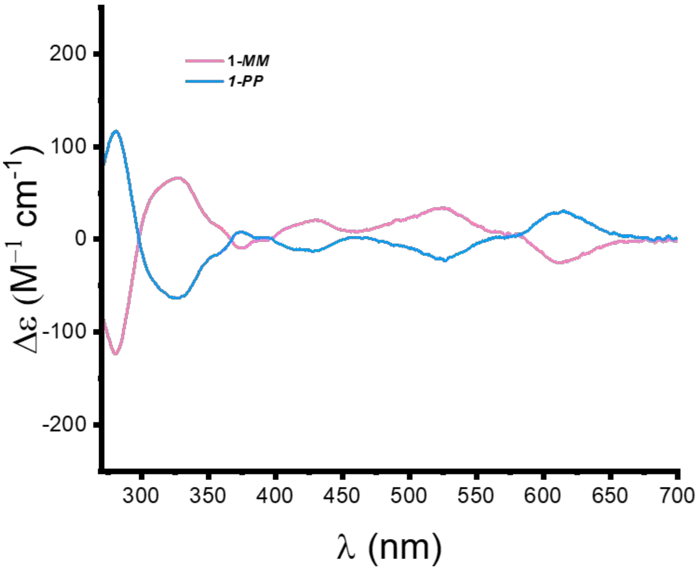

Figure S10: CD spectra for the **1-*MM*** and **1-*PP*** enantiomers of macrocycle **1** in 1,1,2,2-tetrachloroethane (TCE).

b) Circularly polarised luminescence

CPL was measured with a home-built (modular) spectrometer.[9] The excitation source was a broad band (200 – 1000 nm) laser- driven light source EQ 99 (Elliot Scientific). The excitation wavelength was selected by the incorporation of an Acton SP-2155 monochromator (Princeton Instruments); the collimated light was focused into the sample holder (1 cm quartz cuvette). Emission was collected perpendicular to the excitation direction with a lens (f = 150 mm). The emission was fed through a photoelastic modulator (PEM) (Hinds Series II/FS42AA) and through a linear sheet polariser (Comar Optics). The light was then focused into a second scanning monochromator (Acton SP-2155) and subsequently on to a photomultiplier tube (PMT) (Hamamatsu H10723 series). The detection of the CPL signal was achieved using the field modulation lock-in technique. The electronic signal from the PMT was fed into a lock-in amplifier (Hinds Instruments Signaloc Model 2100). The reference signal for the lock-in detection was provided by the PEM control unit. The monchromators, PEM control unit and lock-in amplifier were interfaced to a desktop PC and controlled by a custom-written Labview graphic user interface. The lock-in amplifier provided two signals, an AC signal corresponding to (I_L- I_R) and a DC signal corresponding to (I_L + I_R). Background subtraction was achieved post data collection The emission dissymmetry factor was, therefore, readily obtained from the experimental data as 2 AC/DC.

Spectral calibration of the scanning monochromator was performed using a Hg-Ar calibration lamp (Ocean Optics HL-3P-CAL). A correction factor for the wavelength dependence of the detection system was constructed using a calibrated lamp (Ocean Optics HL-3_CAL). The measured raw data was subsequently corrected using this correction factor. The validation of the CPL detection systems was achieved using light emitting diodes (LEDs) at various emission wavelengths. The LED was mounted in the sample holder and the light from the LED was fed through a broad band polarising filter and $\lambda/4$ plate (Comar Optics) to generate circularly polarised light. Prior to all measurements, the $\lambda/4$ plate and a LED were used to set the phase of the lock-in amplifier correctly. The emission spectra were recorded with 0.5 nm step size and the slits of the detection monochromator were set to a slit width corresponding to a spectral resolution of 0.25 nm. CPL spectra (as well as total emission spectra) were obtained through an averaging procedure of several scans.

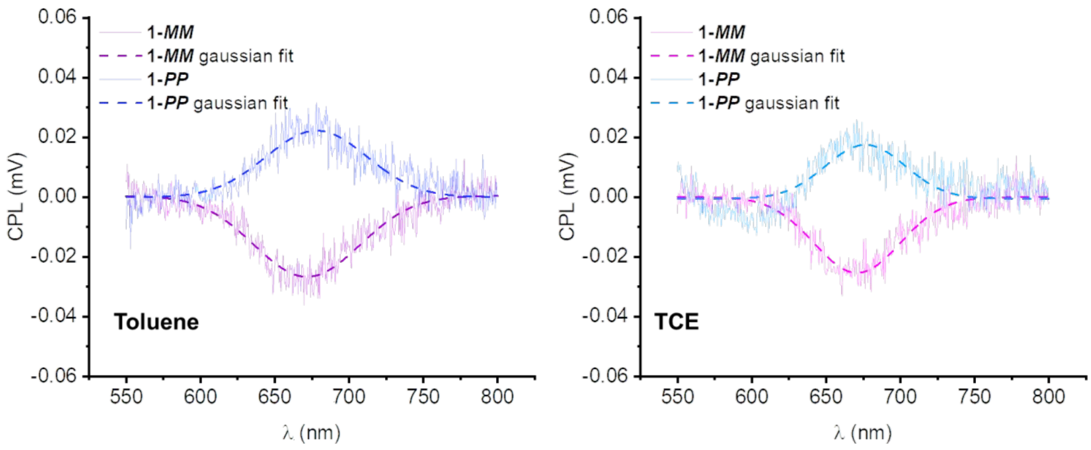

Figure S11: CPL spectra for the **1-*MM*** and **1-*PP*** enantiomers of macrocycle **1** in toluene and TCE (10 μM, 298 K, λ_{ex} = 520 nm).

c) Dissymmetry factors

Table S1: Absorption and emission dissymmetry factors g_{abs} and g_{lum} for macrocycle **1** enantiomers in toluene and TCE (both at 10 μM).

Enantiomer	Solvent	g_{abs} (at 610 nm)	g_{lum} [nm]
MM	Toluene	$- 2.7 \times 10^{-3}$	$- 1.8 \times 10^{-2}$ [672]
PP	Toluene	$+ 2.8 \times 10^{-3}$	$+ 1.7 \times 10^{-2}$ [678]
MM	TCE	$- 2.5 \times 10^{-3}$	$- 1.6 \times 10^{-2}$ [671]
PP	TCE	$+ 2.8 \times 10^{-3}$	$+ 1.5 \times 10^{-2}$ [676]

d) Comparison of macrocycle 1 with other configurationally stable small organic CPL emitters

Figure S12 shows that macrocycle **1-*MM/PP*** has one of the highest g_{lum} values for configurationally stable discrete small organic emitters in solution ($g_{lum} = 2 \times 10^{-2}$) that emit in the red region of the spectrum.

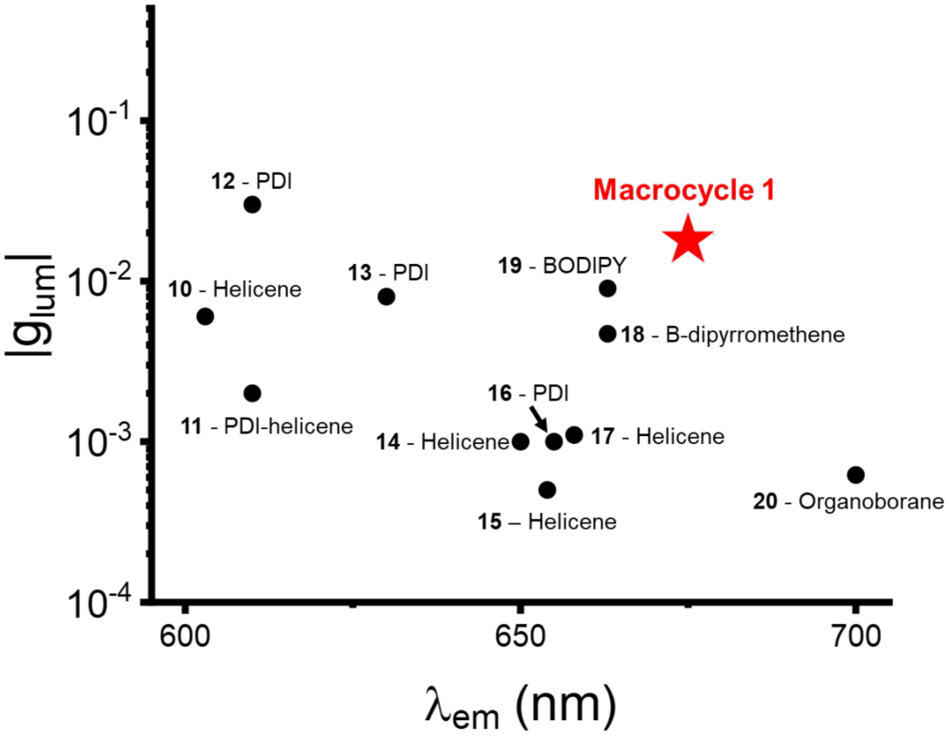

Figure S12: Plot of g_{lum} values against wavelength of emission maxima for a selection of the best performing chirally-locked small organic molecular CPL emitters in solution in the red region of the spectrum (600-700 nm).[10-20]

e) Optical rotation measurements

The specific optical rotations of **1-*MM/PP*** were measured on a Bellingham+Stanley ADP450 digital polarimeter fitted with a 10 cm path length sample holder and a light source with 589 nm wavelength. Measurements were carried out at 20°C and at a concentration of 3 x 10^{-5} g/mL. We measured specific optical rotations $[\alpha]_{\mathrm{D}}^{20}$ of +1333° and –1333° for **1-*MM*** and **1-*PP*** respectively.

5. Photophysics

a) UV-vis-NIR absorption and emission spectra in solution

All steady state electronic absorption and emission spectra were recorded at a concentration of 10 µM (unless otherwise stated) at 298 K. For UV-vis-NIR spectroscopy a Shimadzu UV-3600i Plus spectrophotometer was used, with a wavelength accuracy ± 0.2 nm in the UV-vis range and absorbance accuracy ± 0.002 Abs. For fluorescence spectroscopy a Jasco FP-8500 was used with emission and excitation wavelength accuracies ± 1.0 nm. The detector base sensitivity is 8500:1. Quartz cuvettes with 1 cm path length were used.

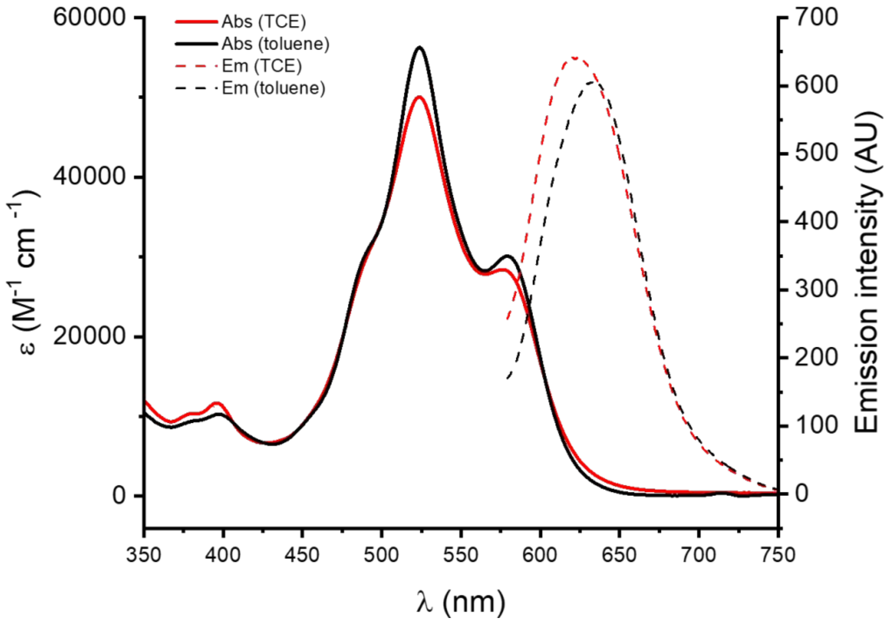

Figure S13: UV-vis absorption and fluorescence emission spectra of macrocycle **1-*rac*** in toluene and TCE (10 µM).

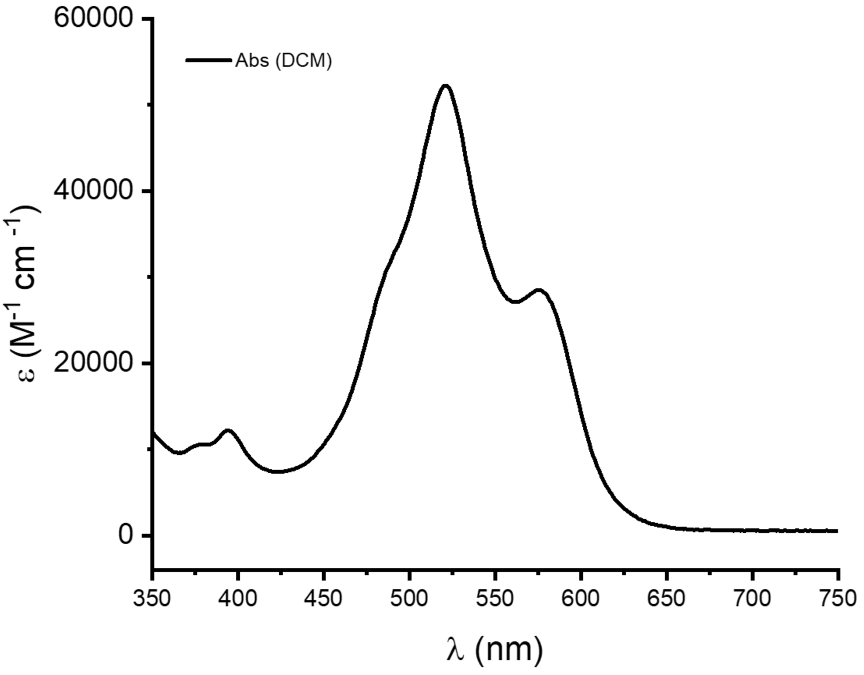

Figure S14: UV-vis absorption spectrum of macrocycle **1-*rac*** in dichloromethane (10 µM).

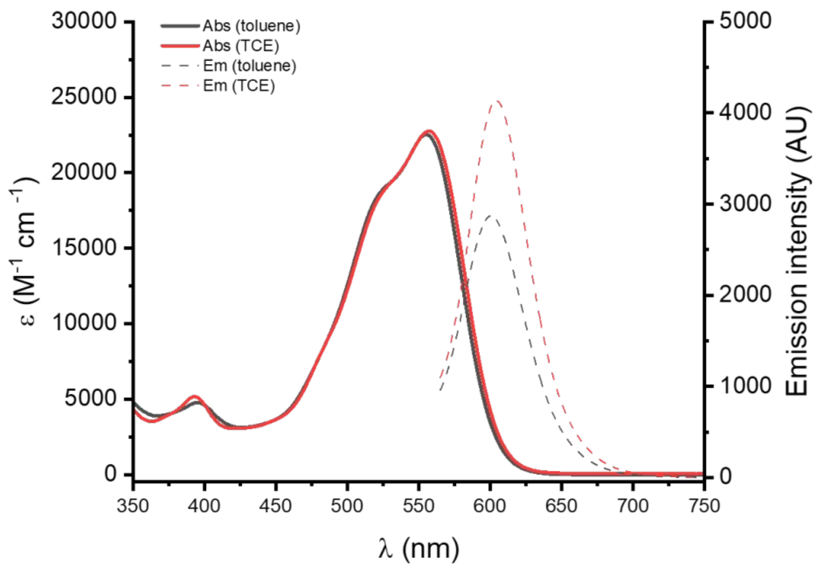

Figure S15: UV-vis absorption and fluorescence emission spectra of acyclic PDI **3** in toluene and TCE (10 μM).

b) Calculation of the exciton coupling energy of macrocycle 1

The exciton coupling energy of macrocycle **1-*rac*** was calculated following the method originally reported by Spano,[21] and used by Würthner to investigate long- and short-range coupling in PDI dimers.[22]

For small rotational displacements of chromophores (< 45°, as seen in macrocycle **1** in the x-ray crystal structure in Figure S7) the rotational displacement of transition dipole moments of two chromophores can be neglected and the ratio of intensities of the 0–0 and 0–1 vibronic absorption bands of a dimer aggregate exhibiting exciton-vibrational coupling can be calculated using the equation:

$$\frac{I_A^{(0-0)}}{I_A^{(0-1)}} = \frac{1}{\lambda^2}\left[\frac{1 - G(0,\lambda^2)e^{-\lambda^2}J/\omega_0}{1 - G(1,\lambda^2)e^{-\lambda^2}J/\omega_0}\right], \quad \omega_0, J \ll |E_{CT} - E_{S1}| \quad [1]$$

Where λ^2 is the Huang-Rhys factor, J is the exciton coupling energy, and ω_0 is the vibrational frequency.

The vibrational function $G(\nu_t,\lambda^2)$ is given by:

$$G(\nu_t,\lambda^2) = \sum_{\substack{u=0,1,\ldots \\ (u \neq \nu_t)}} \frac{\lambda^{2u}}{u!(u-\nu_t)}. \quad \nu_t = 0,1,2,\ldots \quad [2]$$

From the UV-vis absorption spectrum of **1-*rac*** in toluene we measure:

$$\frac{I_A^{(0-0)}}{I_A^{(0-1)}} = 0.567 \quad [3]$$

We obtained the Huang-Rhys factor by gaussian fitting of the vibronic peaks in the UV-vis spectrum of **1-*rac*** (Figure S16), considering only the area of the spectrum arising from the S_0–S_1 transition (< 23360 cm^{-1}). We calculated the Huang-Rhys factor as:

$$\lambda^2 = \frac{I_{\text{Gaussian}}^{(0-1)}}{I_{\text{Gaussian}}^{(0-0)}} = 1.598 \quad [4]$$

The frequency ω_0 was set as 1709 cm^{-1}, corresponding to the energy difference between the maxima of the Gaussian functions of the 0–0 and 0–1 absorption bands. The exciton coupling energy J was then calculated using equation 1. We obtained J = 420.4 cm^{-1}.

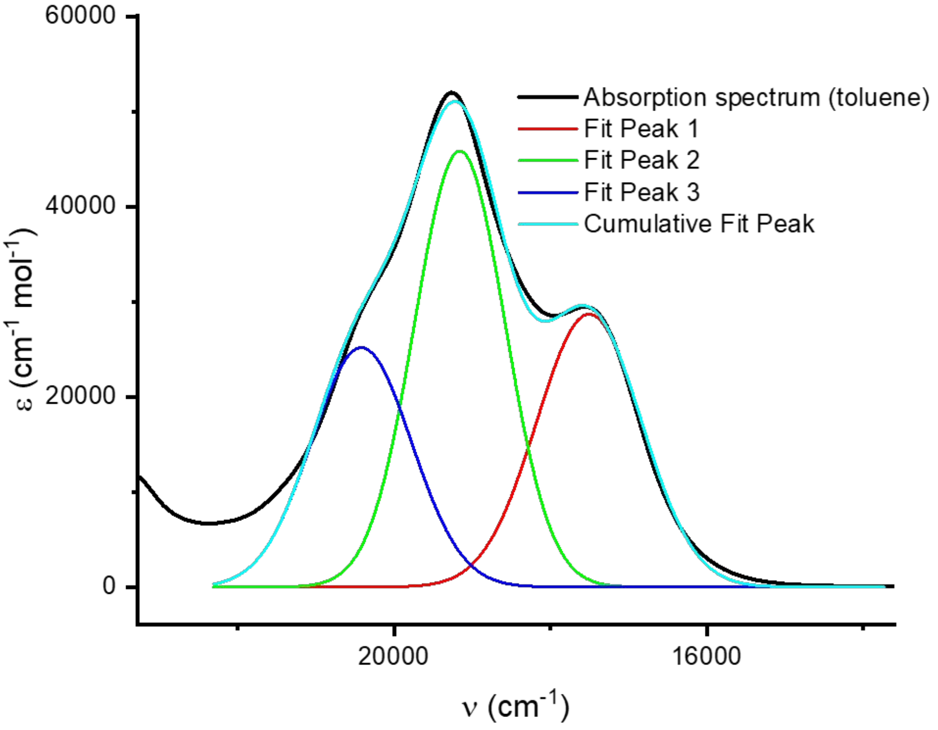

Figure S16: Normalised UV-vis absorption spectrum of macrocycle **1** in toluene (10 µM, black trace), along with the cumulative fit of the spectrum as the sum of three gaussian functions for the 0-0, 0-1 and 0-2 vibronic peaks.

c) Quantum yields

Absolute fluorescence quantum yields were obtained on an Edinburgh Instruments FLS1000 photoluminescence spectrometer fitted with an integrating sphere. All samples were recorded in toluene at a 1 µM with a 7 - 8 nm excitation slit and 0.1 - 0.2 nm emission slit width. Experiments were carried out in solution using 1 cm path length quartz cuvettes with four transparent polished faces.

Table S2: Quantum yields for compounds **1** and **3** in toluene.

Compound	Quantum yield (%)
**1-*rac***	35
**3**	70

d) Fluoresence lifetime measurements

Fluorescence lifetimes measurements were carried out on the emission band of compounds **1-*rac*** (635 nm) and **3** (604 nm) in toluene using a Horiba Fluorolog-3 Time Correlated Single Photon Counting (TCSPC) module controlled by Datastation software. The sample was excited using a 373-nm Delta Diode 373 nm laser attenuated with a neutral density filter and

analysed using DAS6 software. Fluoresence lifetimes were obtained by fitting the resulting data to exponential decay curves.

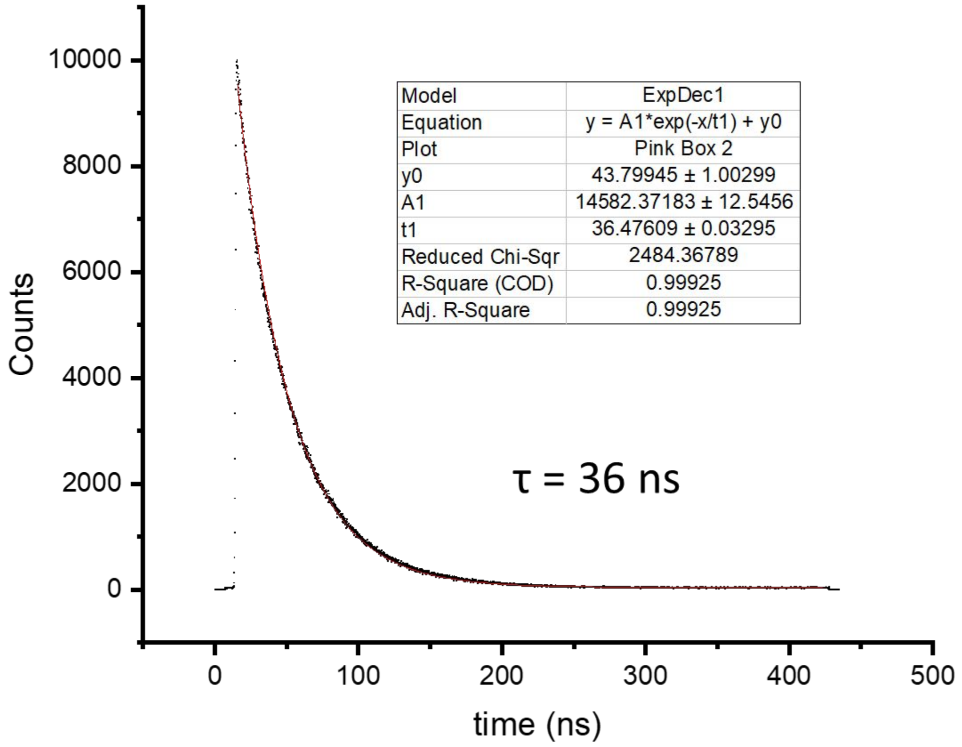

Figure S17: Exponential decay of emission for macrocycle **1-*rac*** (toluene, 5 μmol) measured at 635 nm, from which a fluoresence lifetime of 36 ns is obtained.

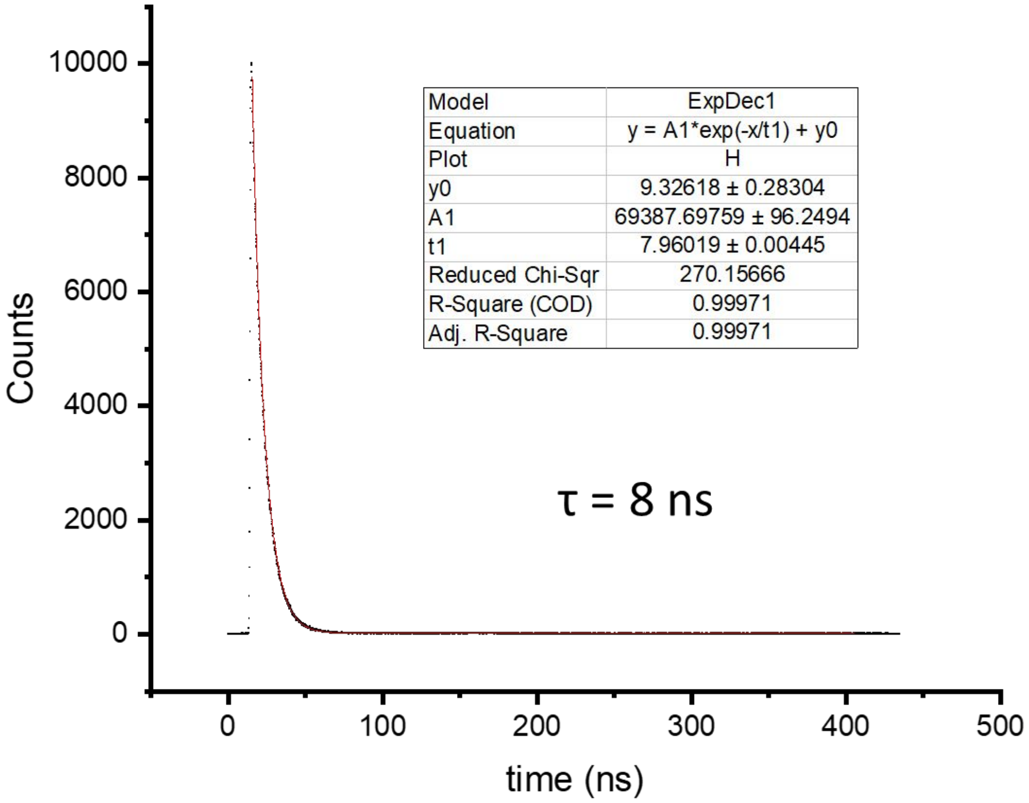

Figure S18: Exponential decay of emission for compound **3** (toluene, 5 μmol) measured at 604 nm, from which a fluoresence lifetime of 8 ns is obtained.

e) UV-vis-NIR absorption (reflectance) and emission spectra from crystals

UV-vis-NIR absorption (reflectance) spectra from crystals of **1-*rac*** and **1-*PP*** were measured on a Shimadzu UV3600i spectrometer fitted with a ISR-603 integrating sphere with a powder sample holder. Absorption spectra were calculated from the resulting reflectance spectra using the Kubelka-Munk function: Abs = $(1\text{-}R^2)/2R$ where R is reflectance.[23] The reflectance spectrum of $BaSO_4$ was measured as a baseline with 100% reflectance.

Emission spectra from crystals were measured on a Horiba Fluorolog-3 (L-configuration) equipped with a 450 W Xenon light source, R928P photomultiplier tube and double monochromators. The crystals were placed on a quartz slide on the instrument's slide-holder attachment.

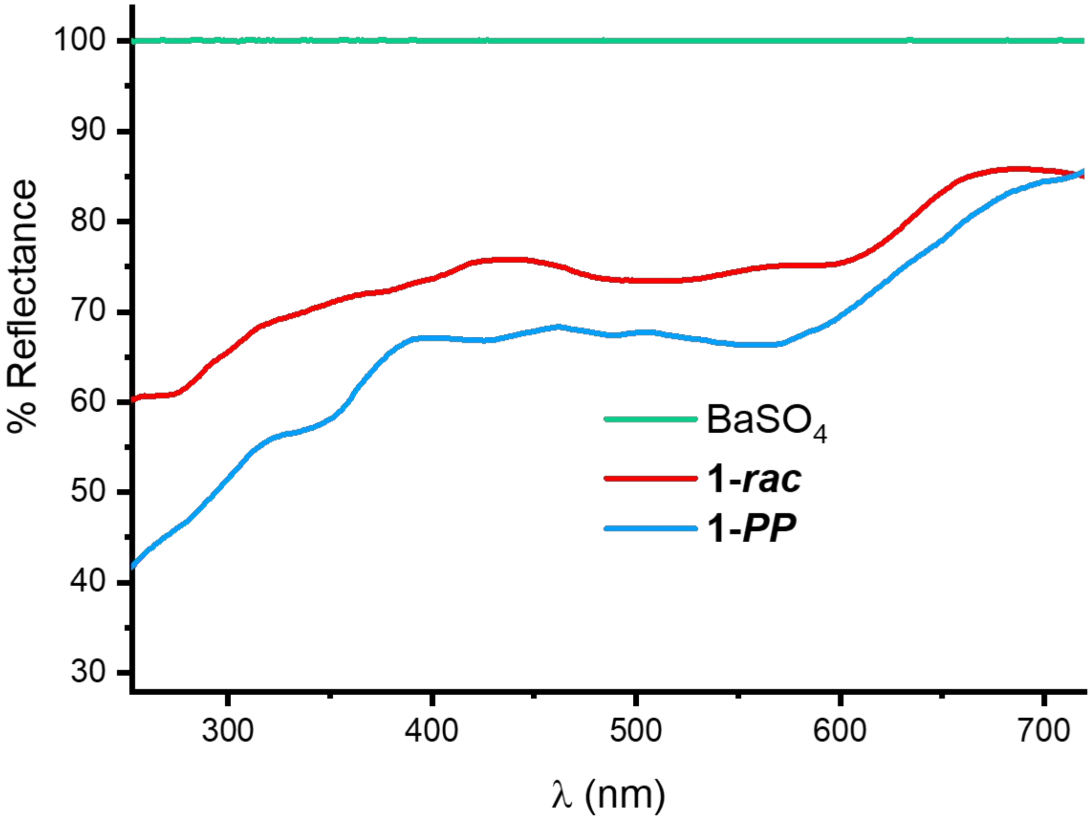

Figure S19: Solid state reflectance spectra for crystals of **1-*rac*** and **1-*PP*** as well as $BaSO_4$.

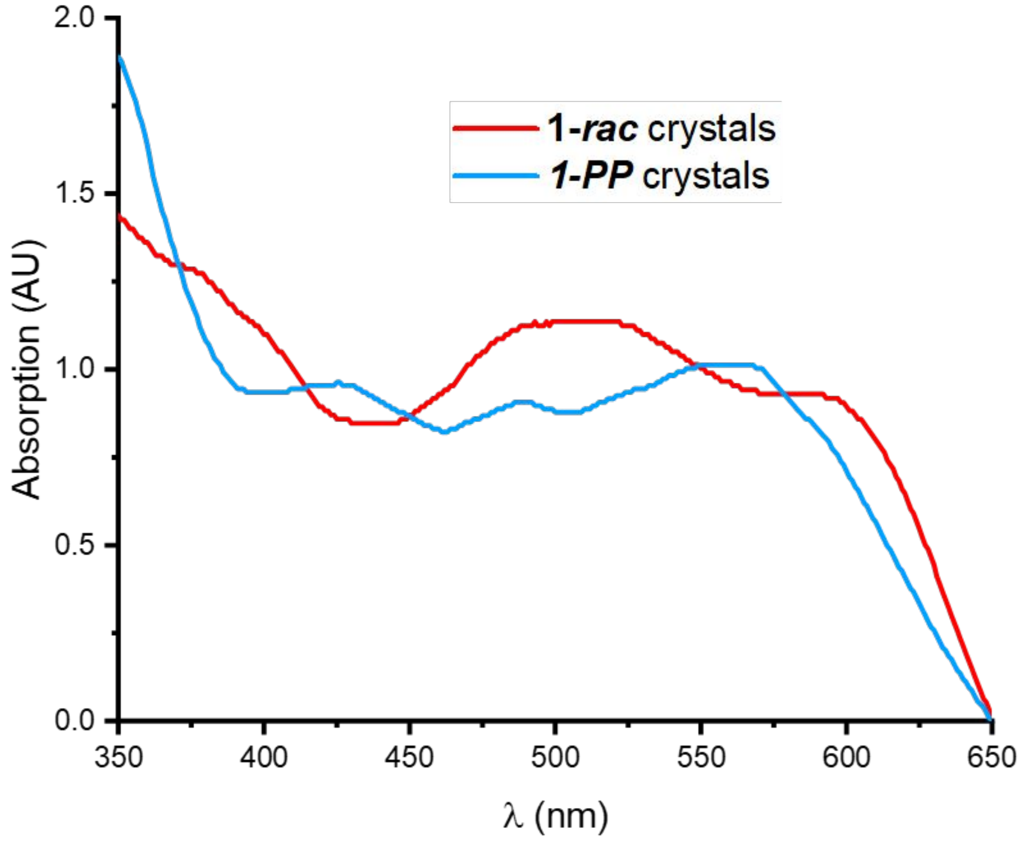

Figure S20: Solid state absorption spectra for crystals of **1-*rac*** and **1-*PP***. Spectra were calculated from raw reflectance spectra using the Kubelka-Munk formula: Abs = $(1-R^2)/2R$ where R is reflectance. The spectra were normalised to give an absorbance of 0 at 650 nm.

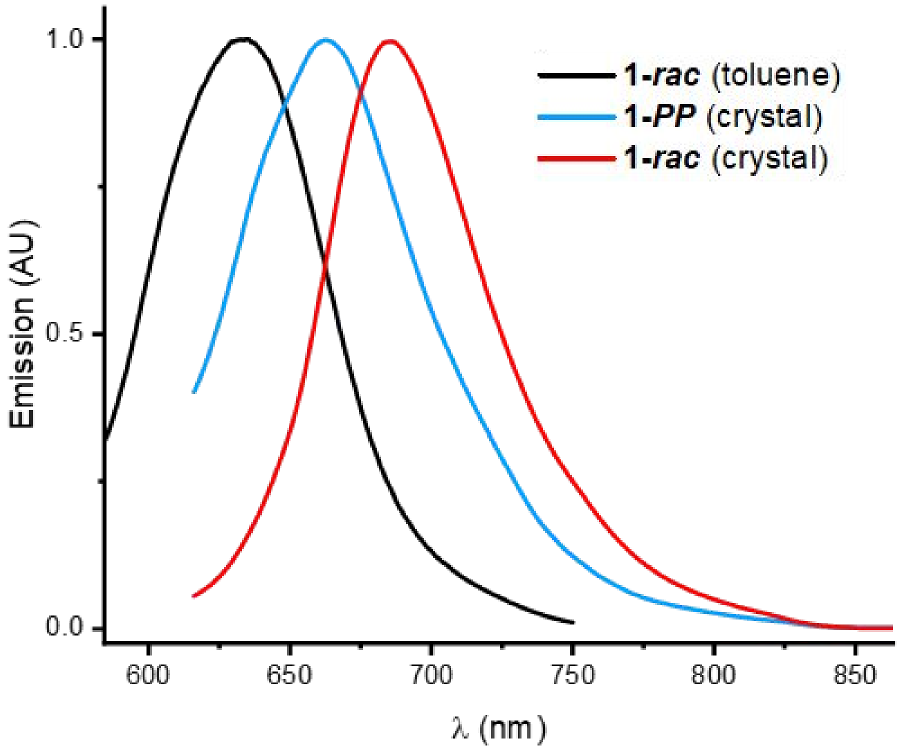

Figure S21: Normalised emission spectra for crystals of **1-*rac*** and **1-*PP*** as well as **1-*rac*** in toluene solution (10 μM).

6. Self-assembly studies

UV-vis spectra were measured as described in Section 5a. Quartz cuvettes with 0.1, 1 and 10 cm path lengths were used to enable accurate spectra to be measured over large concentration ranges (two orders of magnitude). All experiments were carried out in 3:2 CH_2Cl_2:*n*-hexane solution at 298 K. For each experiment, a sample at high concentration (black traces with exact concentration given in Figures S22, S24 and S28) was gradually diluted down to a low concentration (red traces with exact concentration given in Figures S22, S24 and S28) and UV-vis spectra were recorded at all the intermediate concentrations (grey traces). The resulting data sets were then fitted to a range of different binding models (Figures S23, S25-27), with the most appropriate model giving the dimerization and aggregation constants for compounds **1-*rac*** and **3.**

a) Acyclic PDI 3

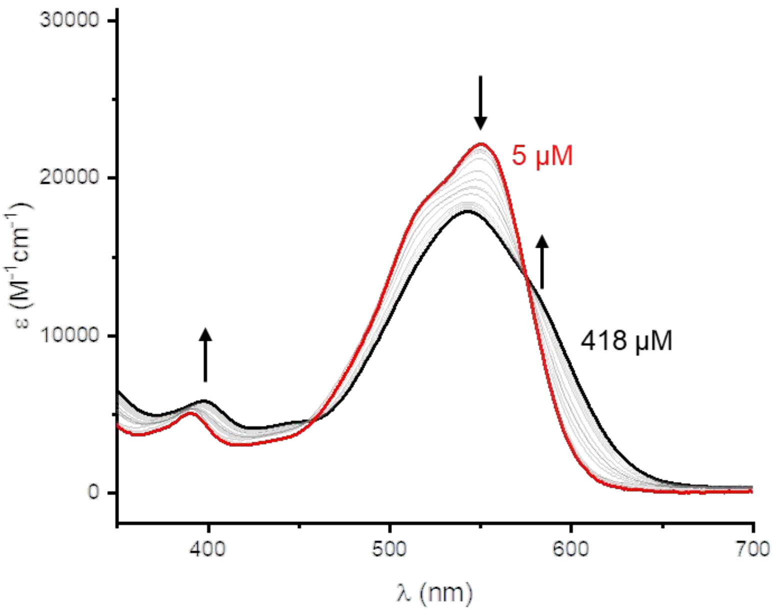

Figure S22: Change in UV-vis absorption spectrum of acyclic PDI **3** upon changing the concentration from 5 μM (red trace) to 418 μM (black trace).

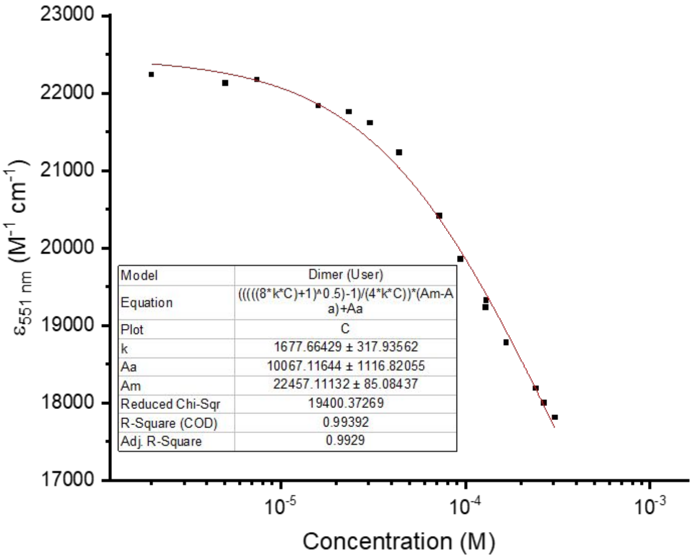

Model	Dimer (User)
Equation	(((((8*k*C)+1)^0.5)-1)/(4*k*C))*(Am-Aa)+Aa
Plot	C
k	1677.66429 ± 317.93562
Aa	10067.11644 ± 1116.82055
Am	22457.11132 ± 85.08437
Reduced Chi-Sqr	19400.37269
R-Square (COD)	0.99392
Adj. R-Square	0.9929

Figure S23: Change in extinction coefficient of acyclic PDI **3** at 551 nm upon changing the concentration from 5 μM to 418 μM, fitted to the monomer-dimer model, giving a dimerization constant K_d = 1678 ± 318 M^{-1}.

b) Macrocycle 1-*rac*

The UV-vis absorption spectrum of racemic macrocycle **1-*rac*** was measured as the sample was concentrated from 0.7 μM to 76 μM (Figure S24). The change in extinction coefficient ε at 596 nm was fitted with various models including the monomer-dimer model (Figure S27), the isodesmic model (Figure S26), and the modified isodesmic (nucleation-elongation) model[24-25] which gave the best fits (Figure S25).

In the modified isodesmic (nucleation-elongation), dimerization (with a distinct dimerization constant $K_d = K_2$) is followed by isodesmic aggregation, such that $K_2 \neq K_3 = K_4 \ldots = K_i \ldots = K$. This can be described by the cubic equation:

$$\alpha_{\mathrm{mon}}{}^3(KC_{\mathrm{T}})^2(\mathrm{p}-1) + \alpha_{\mathrm{mon}}{}^2 KC_{\mathrm{T}}(KC_{\mathrm{T}} - 2(\mathrm{p}-1)) - \alpha_{\mathrm{mon}}(2KC_{\mathrm{T}}+1) + 1 = 0 \qquad [5]$$

Where $\mathrm{p} = K_2/K$. Therefore, $\mathrm{p} < 1$ implies cooperative binding when the aggregate forms after the initial dimerization nucleation event. α_{mon} is the mole fraction of the monomer (i.e., the unaggregated macrocycle) and C_T is the total concentration of the sample. This equation cannot be solved for α_{mon} analytically for general cases. However, it is possible to calculate KC_{T} as a function of KC_{mon} for specific values of p using the equation:

$$KC_T = (1-\mathrm{p})\, KC_{\mathrm{mon}} + \frac{\mathrm{p}KC_{\mathrm{mon}}}{(1-KC_{\mathrm{mon}})^2} \qquad [6]$$

From this we can calculate α_{mon} and α_{agg} (the mole fraction of macrocycle **1-*rac*** in the aggregate state) because:

$$\alpha_{\mathrm{agg}} = 1 - \alpha_{\mathrm{mon}} = 1 - \frac{KC_{\mathrm{mon}}}{KC_{\mathrm{T}}} \qquad [7]$$

α_{agg} is related to the extinction coefficients of the aggregate ($\varepsilon_{\mathrm{agg}}$), monomer ($\varepsilon_{\mathrm{mon}}$) and intermediate state ($\varepsilon(C_{\mathrm{T}})$) by the equation:

$$\alpha_{\mathrm{agg}} = 1 - \frac{\varepsilon(C_{\mathrm{T}}) - \varepsilon_{\mathrm{agg}}}{\varepsilon_{\mathrm{mon}} - \varepsilon_{\mathrm{agg}}} \qquad [8]$$

Hence,

$$\varepsilon(C_{\mathrm{T}}) = \frac{KC_{\mathrm{mon}}}{KC_{\mathrm{T}}}(\varepsilon_{\mathrm{mon}} - \varepsilon_{\mathrm{agg}}) + \varepsilon_{\mathrm{agg}} \qquad [9]$$

Hence the data $\varepsilon(C_{\mathrm{T}})$ can be plotted against KC_{T} and fitted manually to the parameters K, $\varepsilon_{\mathrm{mon}}$, $\varepsilon_{\mathrm{agg}}$ and p.

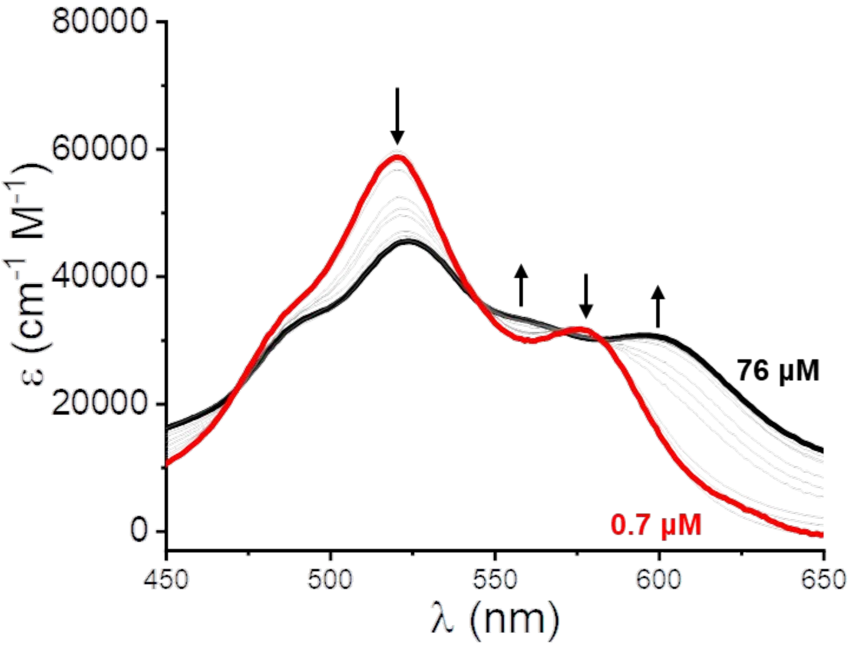

Figure S24: Change in UV-vis absorption spectrum of macrocycle **1-*rac*** upon changing the concentration from 0.7 μM (red trace) to 76 μM (black trace).

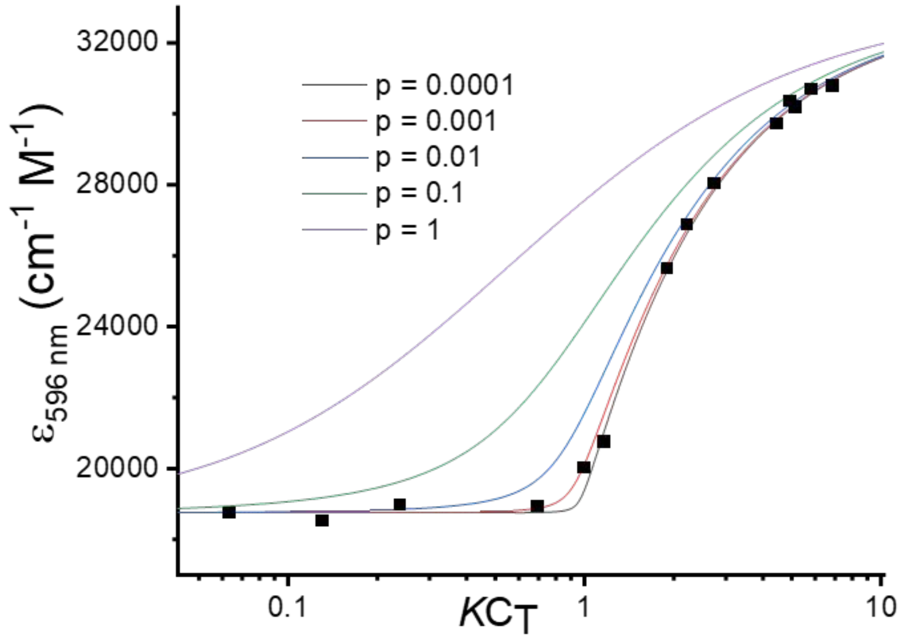

Figure S25: Change in extinction coefficient at 596 nm of macrocycle **1-*rac*** upon changing the concentration from 0.7 μM to 76 μM, manually fitted to the modified isodesmic (nucleation-elongation) model for various values of p = K_2/K. The best manual fit (red trace) was obtained for K = 90,000 M^{-1}, p = 0.001, ε_{agg} = 33,000 cm^{-1} M^{-1} and ε_{mon} = 18,750 cm^{-1} M^{-1}. From this we can deduce K_2 = 90 M^{-1}. We estimate an error of < 10% based on the quality of other fits that we tested.

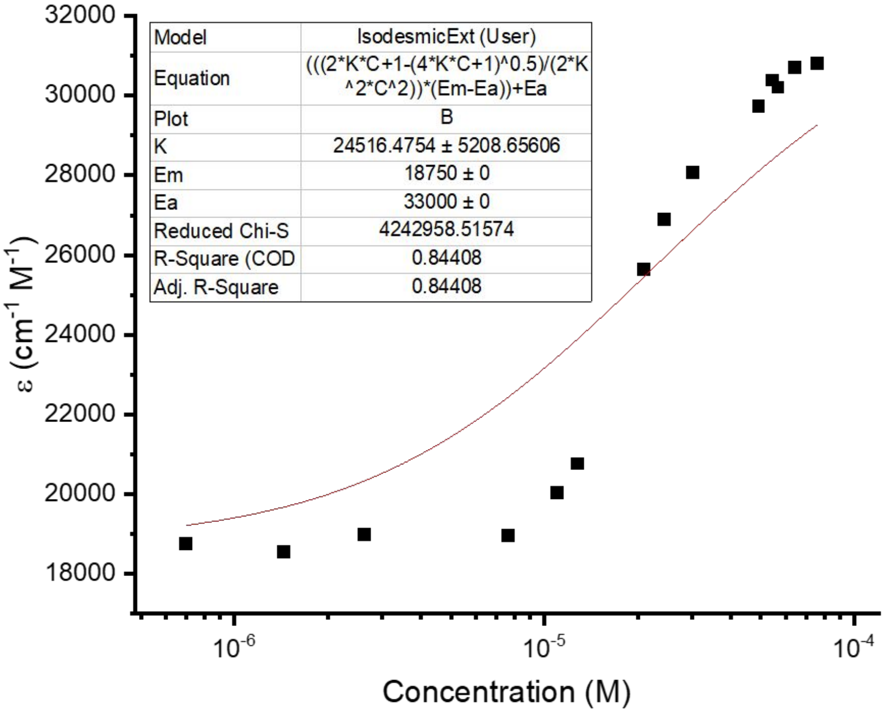

Figure S26: Change in extinction coefficient ε at 596 nm of macrocycle **1-*rac*** upon changing the concentration from 0.7 μM to 76 μM, fitted to the isodesmic model by non-linear regression analysis, with fixed ε_{agg} = 33,000 cm^{-1} M^{-1} and ε_{mon} = 18750 cm^{-1} M^{-1}. The fit is very poor (error ~ 20%). The sudden, steep rise in ε at 10^{-5} M suggests that aggregate formation is non-isodesmic and cooperative. Beer-lambert behaviour is observed below 10^{-5} M.

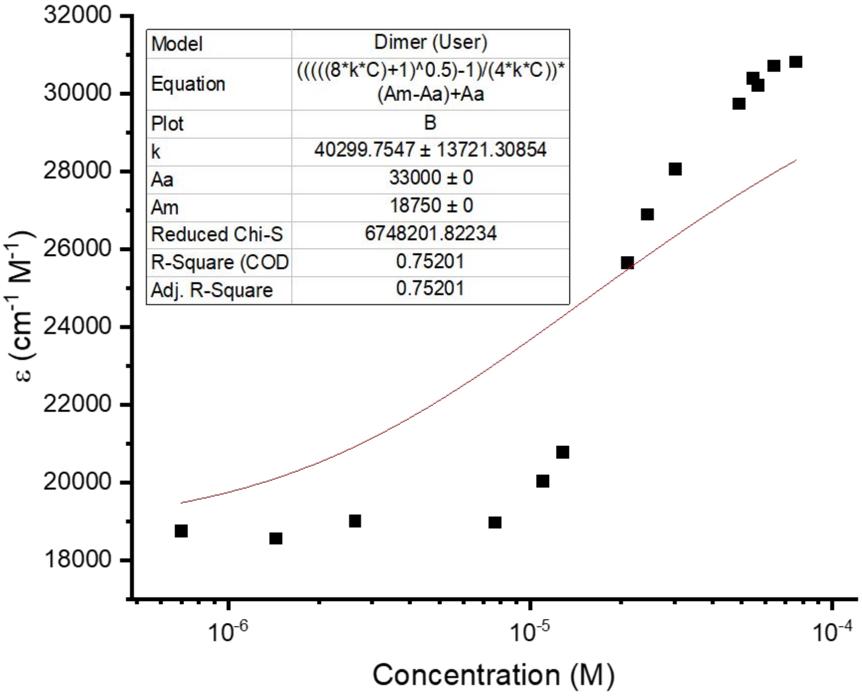

Figure S27: Change in extinction coefficient ε at 596 nm of macrocycle **1-*rac*** upon changing the concentration from 0.7 μM to 76 μM, fitted to the monomer-dimer mode by linear regression analysis, with fixed ε_{agg} = 33,000 cm^{-1} M^{-1} and ε_{mon} = 18750 cm^{-1} M^{-1}. The fit is very poor (error ~ 35%). The sudden, steep rise in ε at 10^{-5} M suggests that aggregate formation is non-isodesmic and cooperative. Beer-lambert behaviour is observed below 10^{-5} M.

c) Macrocycle 1-*PP*

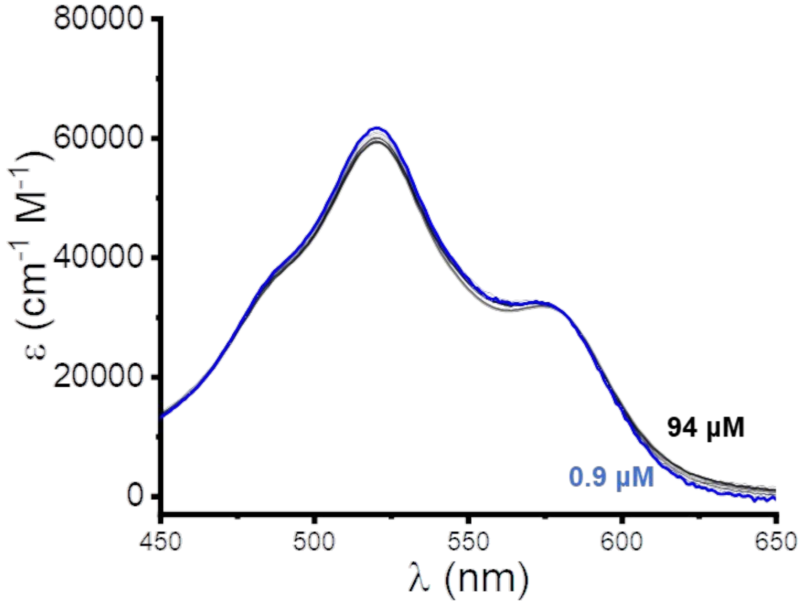

Figure S28: UV-vis absorption spectrum of macrocycle **1-*PP*** upon changing the concentration from 0.9 µM (blue trace) to 94 µM (black trace). The change in the spectrum is negligible compared to **1-*rac***.

d) Attempt to disrupt the *intra*molecular dimer in macrocycle 1

We attempted to disrupt the macrocycle intramolecular dimer by adding methanol to break any hydrogen bonds between the PDI units. However, no significant change to either the UV-vis or NMR spectra is observed on going up to 15% MeOH in TCE.

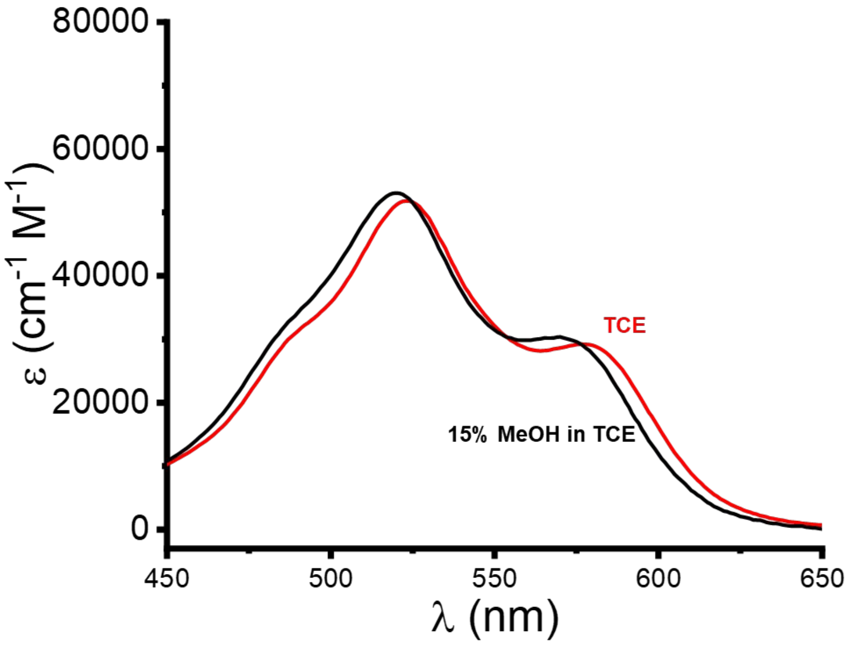

Figure S29: UV-vis absorption spectrum of macrocycle **1-*rac*** in TCE and 15:85 MeOH:TCE. There is no change in the $\varepsilon_{0-0}/\varepsilon_{0-1}$ ratio (0.56 for both spectra), indicating no disruption of the PDI-PDI intramolecular dimer upon adding methanol.

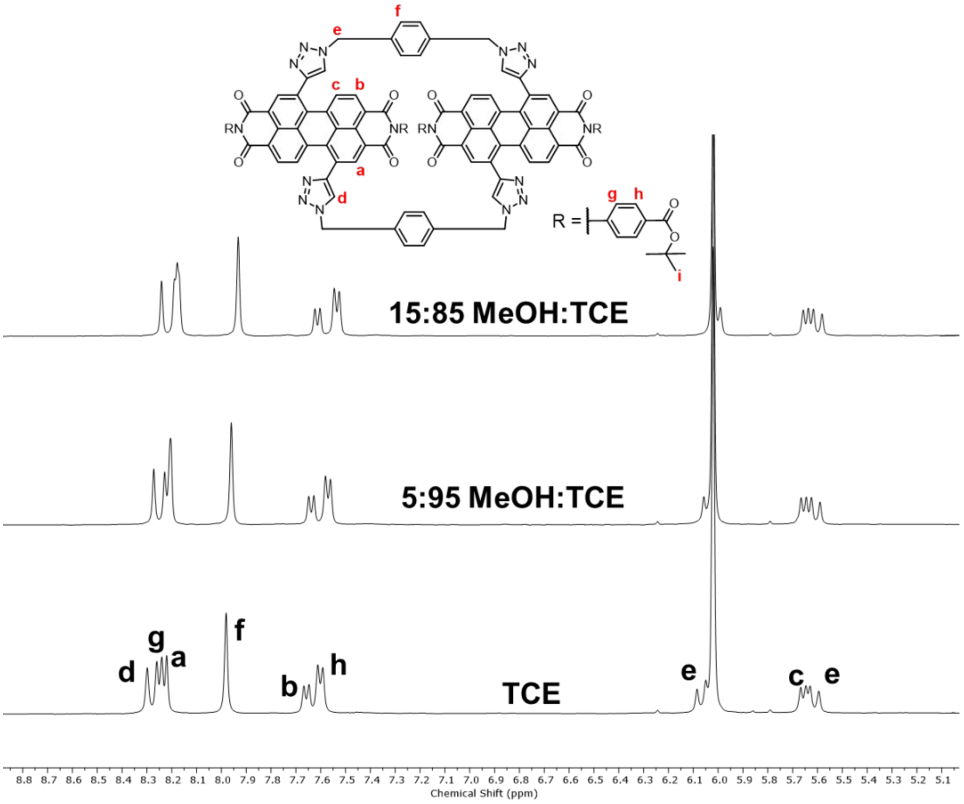

Figure S30: NMR spectra of macrocycle **1-*rac*** in different MeOH:TCE solvent mixtures. No significant change is observed upon increasing the methanol content of the solution.

7. CPL microscopy

a) Circular Polarisation Luminescence Laser Scanning Confocal Microscope (CPL-LSCM)

CPL-LCSM[26] was enabled by adapting a commercial LSCM (SP5 II, Leica Microsystems) with excitation provided by a fibre coupled 80 mW variable power 355 nm Nd:YAG CW laser. The CPL analysis module was external to an output port and all elements were mounted in a 30 mm cage mount system for optimal alignment (assorted 30 mm components, Thorlabs). First, light from the sample focal plane excites the mirror controlled X1 emission port and passes through a selectable high transmission 570 nm longpass filter (FGL570, Thorlabs) mounted in a switchable filter selector apparatus (CFS1/M, Thorlabs). The circularly polarised emission is then converted to linearly polarised light by an achromatic quarter wave plate (AQWP05M-600, Thorlabs) and is separated into two detection arms by a simple 50/50 beam-splitter cube (BS013, Thorlabs). In each arm, the linearly polarised light is selectively analysed by linear polarisers (LPVISE100-A, Thorlabs) mounted within ultra-high precision computer-controlled rotation mounts with ± 60 µrad unidirectional repeatability (K10CR1/M, Thorlabs), orientated to select for left or right CPL states via computer control software (Kinesis, Thorlabs). The intensity of emission in each path was quantified by fibre-coupled (200 micron) high performance matched tandem avalanche photodiodes (Leica ADPs, Becker & Hickl ID-120). The two detection arms were aligned to achieve matched sensitivity to enable rapid simultaneous acquisition of left and right CPL images. Calibration of the linear polarisers for enantioselective localisation was executed based upon the procedure reported by Mackenzie et. al.[27]

b) Enantioselective differential chiral contrast imaging

We performed enantioselective differential chiral contrast (EDCC) imaging of single crystals of **1-*rac*** and **1-*PP/MM*** using CPL-LSCM. In CPL-LSCM, right- and left-circularly polarised photons are collected simultaneously from the sample, generating independent right and left CPL images rapidly. EDDC is used to quantify the difference between the amount of left-handed- and right-handed-circularly polarised photons that are emitted, one of the two images can be subtracted from the other and the brightness of the resulting image determines the chirality-induced helicity dominance of each enantiomer.

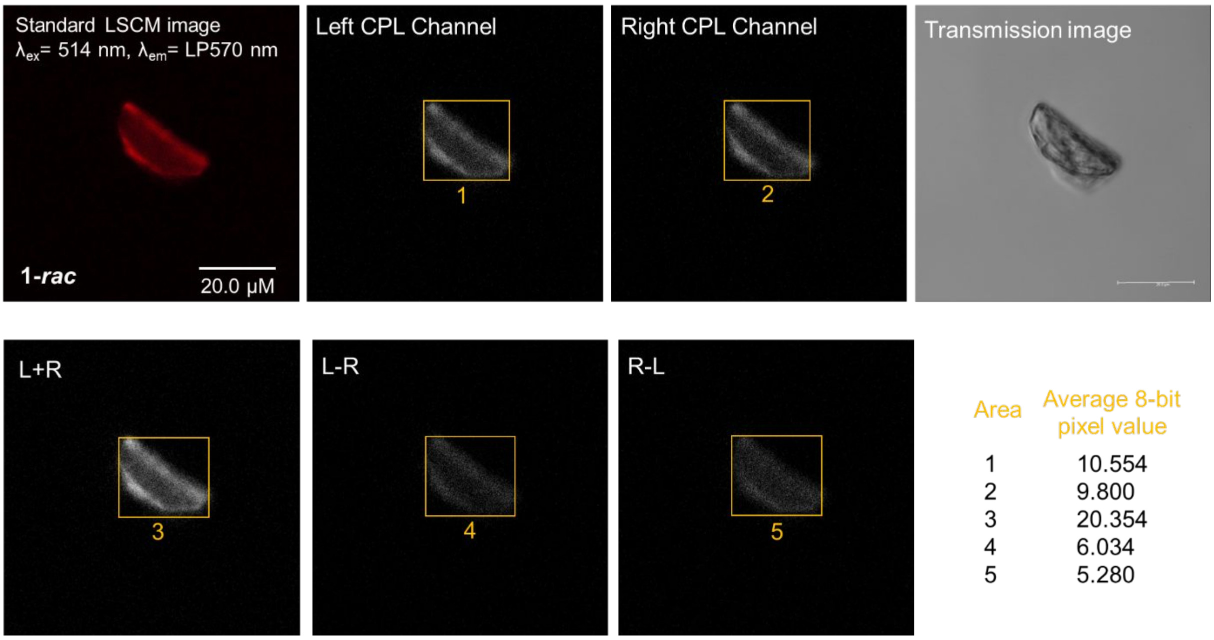

Area	Average 8-bit pixel value
1	10.554
2	9.800
3	20.354
4	6.034
5	5.280

Figure S31: Enantioselective differential chiral contrast (EDCC) images for crystals of **1-*rac*** along with the average 8-bit (0-255 greyscale) pixel values for the highlighted areas in the images.

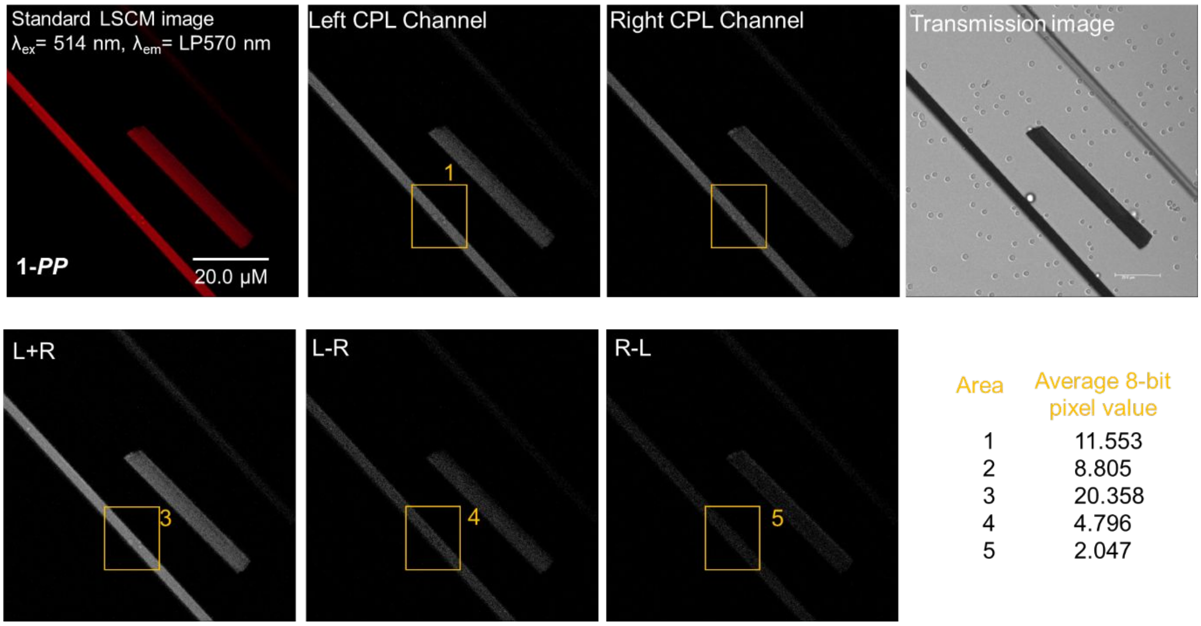

Area	Average 8-bit pixel value
1	11.553
2	8.805
3	20.358
4	4.796
5	2.047

Figure S32: Enantioselective differential chiral contrast (EDCC) images for crystals of **1-*PP*** along with the average 8-bit (0-255 greyscale) pixel values for the highlighted areas in the images.

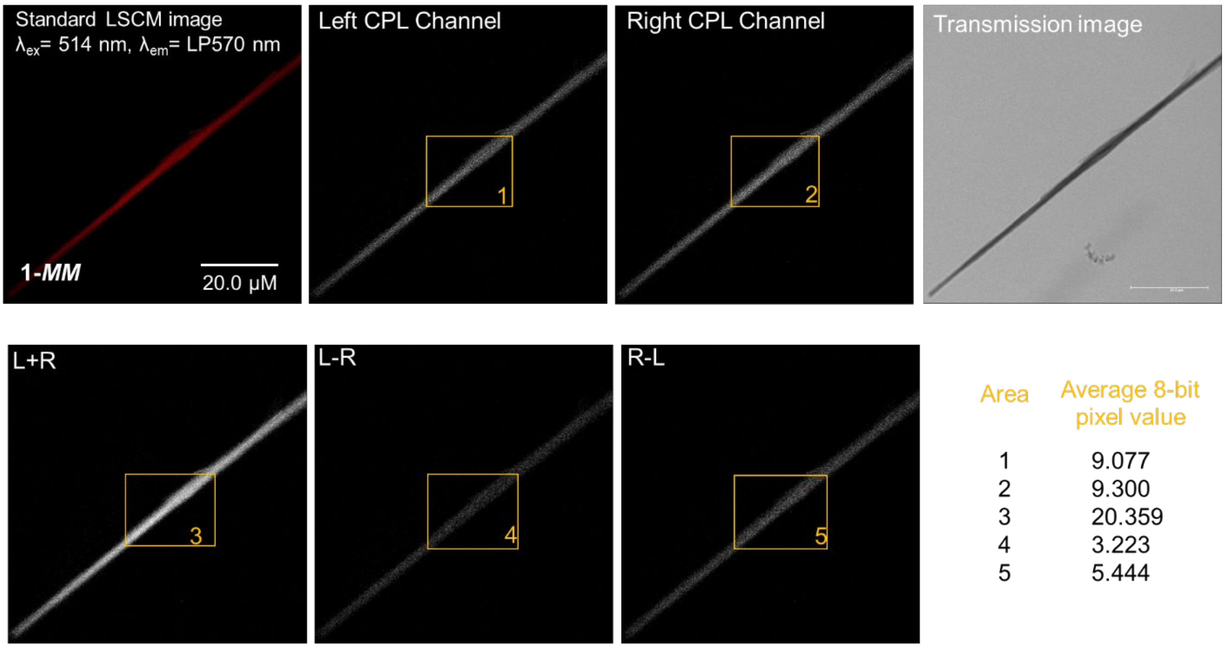

Figure S33: Enantioselective differential chiral contrast (EDCC) images for crystals of **1-*MM*** along with the average 8-bit (0-255 greyscale) pixel values for the highlighted areas in the images.

c) Determination of enantioselective differential chiral contrast dissymmetry factors (g_{EDCC})

Having distinct enantiopure and racemic single crystals provided us with the opportunity to quantify the degree of circularly polarized emitted light from single crystals for the first time using CPL-LSCM, by calculating an EDCC dissymmetry factor (g_{EDCC}), a value analogous to the luminescence dissymmetry factor obtained from CPL spectroscopy (g_{lum}). In doing so, it is critical to correct for the inherent CPL bias arising from orientation induced reflection and helicity inversion of light. Therefore, we calculated a bias factor B as follows:

From the EDCC images of **1-*rac*** we calculate a (left-handed) contrast transfer function (CTF):

$$\mathrm{CTF} = \frac{I_{(L-R)} - I_{(R-L)}}{2} = 0.0185 \qquad [10]$$

Where $I_{(L-R)}$ is the left-handed EDCC average 8-bit pixel value (Left CPL - Right CPL) and $I_{(R-L)}$ is the right-handed EDCC average 8-bit pixel value (Right CPL - Left CPL).

As **1-*rac*** is racemic, it will emit equal amounts of Left- and Right-handed light. From this, the bias factor B is half of the CTF as the bias is present equally in the Left and Right channels, so B = CTF (**1-*rac***) / 2 = 0.0093.

We then define g_{EDCC} as:

$$g_{\mathrm{EDCC}} = \frac{\mathrm{CTF}}{I_{(L+R)}} - \mathrm{B} = \frac{I_{(L-R)} - I_{(R-L)}}{2I_{(L+R)}} - \mathrm{B} \qquad [11]$$

Where $I_{(L+R)}$ is the total image average 8-bit pixel value (Left CPL + Right CPL) and B is the calculated bias factor. From this we obtain g_{EDCC} values of +0.0582 and -0.0643 for the **1-*PP*** and **1-*MM*** crystals respectively.

We can validate this analysis by also calculating the bias factor B from the uncorrected CTFs for the enantiomeric crystals **1-*PP/MM***:

$$\mathrm{B} = \frac{\frac{\mathrm{CTF}\ (\mathbf{1}\text{-}\boldsymbol{PP})}{I_{(L+R)}} + \frac{\mathrm{CTF}\ (\mathbf{1}\text{-}\boldsymbol{MM})}{I_{(L+R)}}}{\sqrt{2}} \qquad [12]$$

From equation [12] we obtain a B value 0.0084, which is within 10% of the B value of 0.0093 calculated from equation [10].

8. Computational studies

Conformer searches for macrocycle **1** were performed using the combination of the CREST code[28] and the GFN2-xTB semiempirical tight-binding method.[29] The lowest energy conformers found using CREST were subsequently reoptimized by means of density functional theory using the B97-3c[30] composite scheme. Solvation effects in the DFT calculations were described using the COSMO[31] (toluene, ε_r 2.83) implicit solvation models. Vertical excitation and circular dichroism spectra of the DFT optimised conformers were calculated by single point calculations using the combination of the ωB97x[32] density functional and the def2-SVP basis-sets.[33] All DFT calculations are performed using Turbomole 7.5.[34-35]

The conformer spectrum of macrocycle **1** was found to be very dense in part because of the degrees of freedom associated with the sidechains on the imide nitrogen. We reoptimized the lowest 40 structures with DFT (B97-3c) and found the structure shown in Figure S34 to be the lowest in energy. As discussed in the main text, it is clear from the structures of the lowest energy conformers that the combination of PDI and the two phenyl rings on either side is too large for them to somersault through the macrocycle cavity.

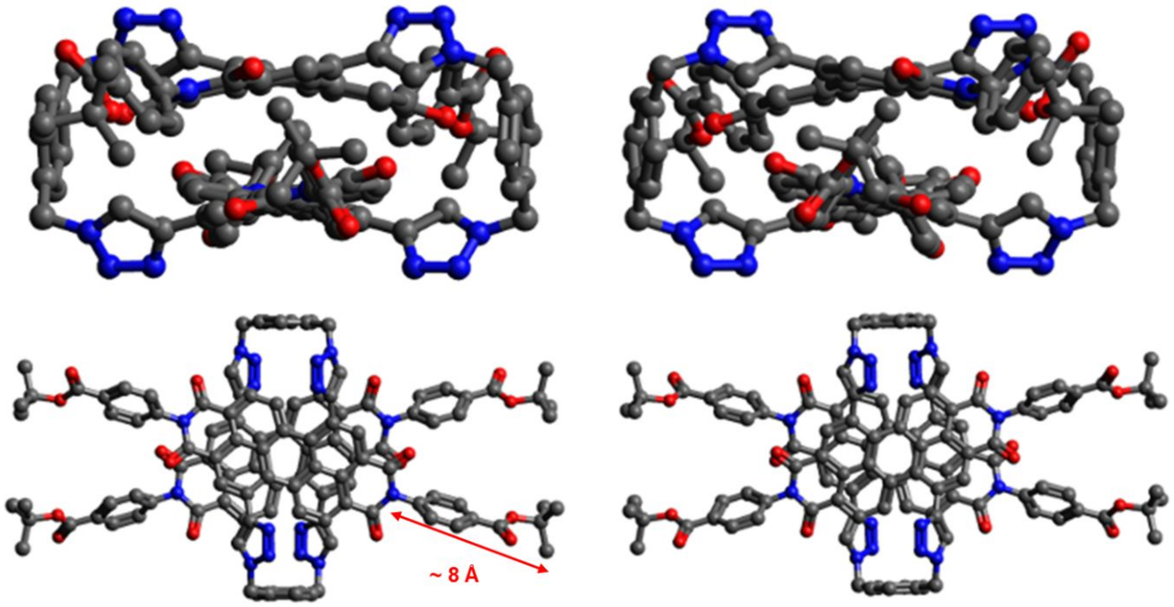

Figure S34: Structures of the ***MM*** (left) and ***PP*** (right) stereoisomers of the lowest energy conformer of macrocycle **1** after B97-3c optimisation (hydrogens omitted for clarity).

Next, we reoptimized the structure of the lowest energy conformer in toluene and predicted the optical absorption spectrum (lowest energy vertical singlet excitations, Table S3) and circular dichroism spectrum (Table S4) in toluene using TD-DFT (wB97x). As expected with use of wB97x the spectrum is blue shifted compared to experiment but can be used to assign the experimental circular dichroism spectrum in Figures 2d and S9 to the ***MM*** and ***PP*** enantiomers of macrocycle **1**.

Table S3: Lowest singlet excitations in toluene as predicted with TD-wB97x for the lowest energy conformer after B97-3c optimisation.

	**Excitation energy / eV**	**Wavelength / nm**	**Oscillator strength / --**
**1**	2.37	522	0.02346
**2**	2.57	482	1.25110
**3**	3.16	392	0.16405
**4**	3.19	389	0.00012
**5**	3.65	339	0.00004
**6**	3.69	336	0.01371

Table S4: Circular dichroism spectrum in toluene as predicted with TD-wB97x for the lowest energy conformer after B97-3c optimisation.

	**Excitation energy / eV (Wavelength / nm)**	**Rotary strength / 10^{-40} erg cm^3**	
		MM	***PP***
**1**	2.37 (522)	-324	+324
**2**	2.57 (482)	+458	-458
**3**	3.16 (392)	-22	+22
**4**	3.19 (389)	-2	2
**5**	3.65 (339)	0	0
**6**	3.69 (336)	+122	-122

9. References

1. Chan, T. R.; Hilgraf, R.; Sharpless, K. B.; Fokin, V. V., Polytriazoles as Copper(I)-Stabilizing Ligands in Catalysis. *Organic Letters* **2004,** *6* (17), 2853-2855.

2. Franceschin, M.; Alvino, A.; Ortaggi, G.; Bianco, A., New hydrosoluble perylene and coronene derivatives. *Tetrahedron Letters* **2004,** *45* (49), 9015-9020.

3. Sheldrick, G. M., SHELXT - integrated space-group and crystal-structure determination. *Acta Crystallogr A Found Adv* **2015,** *71* (Pt 1), 3-8.

4. Sheldrick, G. M., Crystal structure refinement with SHELXL. *Acta Crystallogr C Struct Chem* **2015,** *71* (Pt 1), 3-8.

5. Dolomanov, O. V.; Bourhis, L. J.; Gildea, R. J.; Howard, J. A.; Puschmann, H., OLEX2: a complete structure solution, refinement and analysis program. *Journal of applied crystallography* **2009,** *42* (2), 339-341.

6. Beilsten-Edmands, J.; Winter, G.; Gildea, R.; Parkhurst, J.; Waterman, D.; Evans, G., Scaling diffraction data in the DIALS software package: algorithms and new approaches for multi-crystal scaling. *Acta Crystallogr D Struct Biol* **2020,** *76* (Pt 4), 385-399.

7. Winter, G., xia2: an expert system for macromolecular crystallography data reduction. *Journal of applied crystallography* **2010,** *43* (1), 186-190.

8. Winter, G.; Waterman, D. G.; Parkhurst, J. M.; Brewster, A. S.; Gildea, R. J.; Gerstel, M.; Fuentes-Montero, L.; Vollmar, M.; Michels-Clark, T.; Young, I. D.; Sauter, N. K.; Evans, G., DIALS: implementation and evaluation of a new integration package. *Acta Crystallogr D Struct Biol* **2018,** *74* (Pt 2), 85-97.

9. Carr, R.; Puckrin, R.; McMahon, B. K.; Pal, R.; Parker, D.; Pålsson, L.-O., Induced circularly polarized luminescence arising from anion or protein binding to racemic emissive lanthanide complexes. *Methods and Applications in Fluorescence* **2014,** *2* (2), 024007.

10. Zhao, Z.-H.; Liang, X.; He, M.-X.; Zhang, M.-Y.; Zhao, C.-H., Triarylborane-based [5]Helicenes with Full-Color Circularly Polarized Luminescence. *Organic Letters* **2019,** *21* (23), 9569-9573.

11. Liu, B.; Böckmann, M.; Jiang, W.; Doltsinis, N. L.; Wang, Z., Perylene Diimide-Embedded Double [8]Helicenes. *Journal of the American Chemical Society* **2020,** *142* (15), 7092-7099.

12. Liu, Y.; Ma, Z.; Wang, Z.; Jiang, W., Boosting Circularly Polarized Luminescence Performance by a Double π-Helix and Heteroannulation. *Journal of the American Chemical Society* **2022,** *144* (25), 11397-11404.

13. Kumar, J.; Nakashima, T.; Tsumatori, H.; Mori, M.; Naito, M.; Kawai, T., Circularly Polarized Luminescence in Supramolecular Assemblies of Chiral Bichromophoric Perylene Bisimides. *Chemistry – A European Journal* **2013,** *19* (42), 14090-14097.

14. Dhbaibi, K.; Favereau, L.; Srebro-Hooper, M.; Jean, M.; Vanthuyne, N.; Zinna, F.; Jamoussi, B.; Di Bari, L.; Autschbach, J.; Crassous, J., Exciton coupling in diketopyrrolopyrrole–helicene derivatives leads to red and near-infrared circularly polarized luminescence. *Chemical Science* **2018,** *9* (3), 735-742.

15. Pascal, S.; Besnard, C.; Zinna, F.; Di Bari, L.; Le Guennic, B.; Jacquemin, D.; Lacour, J., Zwitterionic [4]helicene: a water-soluble and reversible pH-triggered ECD/CPL chiroptical

switch in the UV and red spectral regions. *Organic & Biomolecular Chemistry* **2016,** *14* (20), 4590-4594.

16. Renner, R.; Mahlmeister, B.; Anhalt, O.; Stolte, M.; Würthner, F., Chiral Perylene Bisimide Dyes by Interlocked Arene Substituents in the Bay Area. *Chemistry – A European Journal* **2021,** *27* (46), 11997-12006.

17. Bosson, J.; Labrador, G. M.; Pascal, S.; Miannay, F.-A.; Yushchenko, O.; Li, H.; Bouffier, L.; Sojic, N.; Tovar, R. C.; Muller, G.; Jacquemin, D.; Laurent, A. D.; Le Guennic, B.; Vauthey, E.; Lacour, J., Physicochemical and Electronic Properties of Cationic [6]Helicenes: from Chemical and Electrochemical Stabilities to Far-Red (Polarized) Luminescence. *Chemistry – A European Journal* **2016,** *22* (51), 18394-18403.

18. Alnoman, R. B.; Rihn, S.; O'Connor, D. C.; Black, F. A.; Costello, B.; Waddell, P. G.; Clegg, W.; Peacock, R. D.; Herrebout, W.; Knight, J. G.; Hall, M. J., Circularly Polarized Luminescence from Helically Chiral N,N,O,O-Boron-Chelated Dipyrromethenes. *Chemistry – A European Journal* **2016,** *22* (1), 93-96.

19. Saikawa, M.; Nakamura, T.; Uchida, J.; Yamamura, M.; Nabeshima, T., Synthesis of figure-of-eight helical bisBODIPY macrocycles and their chiroptical properties. *Chemical Communications* **2016,** *52* (71), 10727-10730.

20. Zhao, F.; Zhao, J.; Liu, H.; Wang, Y.; Duan, J.; Li, C.; Di, J.; Zhang, N.; Zheng, X.; Chen, P., Synthesis of π-Conjugated Chiral Organoborane Macrocycles with Blue to Near-Infrared Emissions and the Diradical Character of Cations. *Journal of the American Chemical Society* **2023,** *145* (18), 10092-10103.

21. Kistler, K. A.; Pochas, C. M.; Yamagata, H.; Matsika, S.; Spano, F. C., Absorption, Circular Dichroism, and Photoluminescence in Perylene Diimide Bichromophores: Polarization-Dependent H- and J-Aggregate Behavior. *The Journal of Physical Chemistry B* **2012,** *116* (1), 77-86.

22. Kaufmann, C.; Bialas, D.; Stolte, M.; Würthner, F., Discrete π-Stacks of Perylene Bisimide Dyes within Folda-Dimers: Insight into Long- and Short-Range Exciton Coupling. *Journal of the American Chemical Society* **2018,** *140* (31), 9986-9995.

23. Makuła, P.; Pacia, M.; Macyk, W., How To Correctly Determine the Band Gap Energy of Modified Semiconductor Photocatalysts Based on UV–Vis Spectra. *The Journal of Physical Chemistry Letters* **2018,** *9* (23), 6814-6817.

24. Kaiser, T. E.; Stepanenko, V.; Würthner, F., Fluorescent J-Aggregates of Core-Substituted Perylene Bisimides: Studies on Structure−Property Relationship, Nucleation−Elongation Mechanism, and Sergeants-and-Soldiers Principle. *Journal of the American Chemical Society* **2009,** *131* (19), 6719-6732.

25. Chen, Z.; Lohr, A.; Saha-Möller, C. R.; Würthner, F., Self-assembled π-stacks of functional dyes in solution: structural and thermodynamic features. *Chemical Society Reviews* **2009,** *38* (2), 564-584.

26. Stachelek, P.; MacKenzie, L.; Parker, D.; Pal, R., Circularly polarised luminescence laser scanning confocal microscopy to study live cell chiral molecular interactions. *Nature Communications* **2022,** *13* (1), 553.

27. MacKenzie, L. E.; Pålsson, L.-O.; Parker, D.; Beeby, A.; Pal, R., Rapid time-resolved Circular Polarization Luminescence (CPL) emission spectroscopy. *Nature Communications* **2020,** *11* (1), 1676.

28. Pracht, P.; Bohle, F.; Grimme, S., Automated exploration of the low-energy chemical space with fast quantum chemical methods. *Physical Chemistry Chemical Physics* **2020,** *22* (14), 7169-7192.

29. Bannwarth, C.; Ehlert, S.; Grimme, S., GFN2-xTB—An Accurate and Broadly Parametrized Self-Consistent Tight-Binding Quantum Chemical Method with Multipole Electrostatics and Density-Dependent Dispersion Contributions. *Journal of Chemical Theory and Computation* **2019,** *15* (3), 1652-1671.

30. Brandenburg, J. G.; Bannwarth, C.; Hansen, A.; Grimme, S., B97-3c: A revised low-cost variant of the B97-D density functional method. *The Journal of Chemical Physics* **2018,** *148* (6).

31. Klamt, A.; Schüürmann, G., COSMO: a new approach to dielectric screening in solvents with explicit expressions for the screening energy and its gradient. *Journal of the Chemical Society, Perkin Transactions 2* **1993,** (5), 799-805.

32. Chai, J.-D.; Head-Gordon, M., Systematic optimization of long-range corrected hybrid density functionals. *The Journal of Chemical Physics* **2008,** *128* (8).

33. Weigend, F.; Ahlrichs, R., Balanced basis sets of split valence, triple zeta valence and quadruple zeta valence quality for H to Rn: Design and assessment of accuracy. *Physical Chemistry Chemical Physics* **2005,** *7* (18), 3297-3305.

34. Furche, F.; Ahlrichs, R.; Hättig, C.; Klopper, W.; Sierka, M.; Weigend, F., Turbomole. *WIREs Computational Molecular Science* **2014,** *4* (2), 91-100.

35. Balasubramani, S. G.; Chen, G. P.; Coriani, S.; Diedenhofen, M.; Frank, M. S.; Franzke, Y. J.; Furche, F.; Grotjahn, R.; Harding, M. E.; Hättig, C.; Hellweg, A.; Helmich-Paris, B.; Holzer, C.; Huniar, U.; Kaupp, M.; Marefat Khah, A.; Karbalaei Khani, S.; Müller, T.; Mack, F.; Nguyen, B. D.; Parker, S. M.; Perlt, E.; Rappoport, D.; Reiter, K.; Roy, S.; Rückert, M.; Schmitz, G.; Sierka, M.; Tapavicza, E.; Tew, D. P.; van Wüllen, C.; Voora, V. K.; Weigend, F.; Wodyński, A.; Yu, J. M., TURBOMOLE: Modular program suite for ab initio quantum-chemical and condensed-matter simulations. *The Journal of Chemical Physics* **2020,** *152* (18).
